# Single-cell transcriptomics of East-Asian pancreatic islets cells

**DOI:** 10.1038/s41598-017-05266-4

**Published:** 2017-07-10

**Authors:** Rajkumar Dorajoo, Yusuf Ali, Vanessa S. Y. Tay, Jonathan Kang, Sudhagar Samydurai, Jianjun Liu, Bernhard O. Boehm

**Affiliations:** 1 Genome Institute of Singapore, Agency for Science Technology and Research, Singapore, Singapore; 2 Lee Kong Chian School of Medicine, Nanyang Technological University, Singapore, Singapore; 3 Singapore Eye Research Institute, The Academia, Singapore, Singapore; 4 Saw Swee Hock School of Public Health, National University of Singapore and National University Health System, Singapore, Singapore; 5 Department of Medicine, Yong Loo Lin School of Medicine, National University of Singapore, Singapore, Singapore; 6 Department of Endocrinology, Tan Tock Seng Hospital, Singapore, Singapore; 7 Imperial College London, London, UK

## Abstract

Single-cell RNA-seq (scRNA-seq) of pancreatic islets have reported on α- and β-cell gene expression in mice and subjects of predominantly European ancestry. We aimed to assess these findings in East-Asian islet-cells. 448 islet-cells were captured from three East-Asian non-diabetic subjects for scRNA-seq. Hierarchical clustering using pancreatic cell lineage genes was used to assign cells into cell-types. Differentially expressed transcripts between α- and β-cells were detected using ANOVA and *in silico* replications of mouse and human islet cell genes were performed. We identified 118 α, 105 β, 6 δ endocrine cells and 47 exocrine cells. Besides *INS* and *GCG*, 26 genes showed differential expression between α- and β-cells. 10 genes showed concordant expression as reported in rodents, while *FAM46A* was significantly discordant. Comparing our East-Asian data with data from primarily European subjects, we replicated several genes implicated in nuclear receptor activations, acute phase response pathway, glutaryl-CoA/tryptophan degradations and EIF2/AMPK/mTOR signaling. Additionally, we identified protein ubiquitination to be associated among East-Asian β-cells. We report on East-Asian α- and β-cell gene signatures and substantiate several genes/pathways. We identify expression signatures in East-Asian β-cells that perhaps reflects increased susceptibility to cell-death and warrants future validations to fully appreciate their role in East-Asian diabetes pathogenesis.

## Introduction

Data suggest that East-Asians may develop Type 2 diabetes (T2D) at a younger age and at lower BMI levels as compared to European ancestry populations^1, 2^. Worryingly, subjects with younger onset and lean diabetes tend to be less likely to achieve metabolic targets and have a higher prevalence of subsequent comorbidities^2^. Genome-wide association studies have successfully uncovered numerous common variants associated with T2D and highlight on inter-ethnic differences in frequency and effect size at these risk loci (for eg. at the *TCF7L2* locus)^3^. Despite these accumulating genetic information, due to modest effect sizes conferred at these common T2D risk loci, major limitations still exists in clearly delineating the disease phenotype observed in East-Asians.

Islet cells are centrally involved in the etiology of diabetes. Ethnic differences in islet cell function may exist due to inherent genetics and epigenetic changes driven by varied lifestyles and is suggested to particularly predispose Asian subjects to T2D^4, 5^. Evaluation of gene expression in target tissues perhaps represents a combined reflection of pure genetic effects and lifestyle and environmental influences and may identify novel pathways associated with disease^6^. Advances in single-cell RNA-seq (scRNA-seq) techniques enable identification of novel transcripts and cellular heterogeneities and very recent studies in mice^7^ and human^8–11^ pancreatic islets have provided novel transcriptomic insights into islet cell-type biology. However, as most human islet scRNA-seq studies have been performed predominantly in subjects of European ancestry, it is unclear if reported gene signatures are transferrable across ethnicities. We performed scRNA-seq on islet cells captured from three non-diabetic Singaporean Chinese subjects and aimed to evaluate for common and unique expression signatures with recent studies^7–11^.

## Methods

### Human islets

Pancreatic islets were obtained from three non-diabetic Singaporean Chinese subjects from the LKCMedicine Islet Isolation Facility that obtains human pancreata through the Singapore National Organ Transplant Unit (Supplementary Table 1). Informed consent was obtained from all subjects, all methods were carried out in accordance with relevant guidelines and regulations and the study was approved by the Institutional Review Board of the Singapore National Organ Transplant Unit (#IRB-2013-09-005). Islets were cultured for 3 days in complete CMRL-1066 media prior to being handpicked under a stereomicroscope for both functional assay (GSIS, Glucose Stimulated Insulin Secretion) and scRNA-seq studies. Islets with hypoxic cores were discarded. Subsequently, handpicked islets were dissociated into single-cells using Accutase® Cell Detachment Solution (Sigma Aldrich, St.Louis, MO, USA) and re-suspended in complete CMRL-1066 media. For GSIS, islets were incubated in 3 mmol/L glucose for one hour before being placed in a perfusion chamber and exposed to 3 mmol/L glucose (Low Glucose) for 10 minutes followed by 16.7 mmol/L glucose (High Glucose) for 10 minutes. These studies confirmed that islets used in this study exhibited normal insulin secretion profiles (Supplementary Table 1).

### Single-cell RNA-seq (scRNA-seq)

Single human islet cells were quantified using an automated cell counter (Bio-Rad TC20™) and single-cell suspension concentrations were adjusted to approximately 200,000 cells/ml prior to cell capture, as recommended (Fluidigm). Dissociated islet cells had a consistent viability of about 95% and were observed with a size range of approximately 8 to 14 µm. Single human islet cells were captured using medium filter chips (10 to 17 µm) on the Fluidigm C1™ Auto-prep system, as previously performed^7–9^. Captured cells in each well of the C1 chip were visually inspected on a Nikon ECLIPSE Ti microscope, fitted with a 96-well C1 chip holder. Wells with no cell captured or with more than one cell captured were excluded (Supplementary Figure 1).

138, 84 and 226 single-cells from subject 1, 2 and 3, respectively (Supplementary Table 1) were processed for RNA-seq using Nextera XT kits (Illumina). Cell lysis, reverse transcription (SMARTer Ultra Low RNA kit, Clontech) and PCR amplification (Advantage® 2 kit, Clontech) were subsequently performed on the C1™ Auto-prep module. cDNA were aliquoted and quantified using picogreen serial dilutions. Approximately 0.15 ng of cDNA from each cell was processed for RNA-seq using Nextera XT DNA library preparation kits (Illumina). cDNA from 63 and 75 single cells from subject 1, 84 single cells from subject 2 and 82, 73 and 71 single cells were pooled into multiplex libraries and sequenced on 1 lane on Hiseq 4000 each.

### scRNA-seq quality control (QC)

Six cells with low sequence reads (<1 million reads) were excluded from analysis. Mean reads in remaining cells was 3.68 million (1.02 million–8.21 million). Sequence reads were aligned to hg19 and mRNA quantified to RPKM values using Partek Genomics Suite (ver6.6). mRNA levels between-samples were normalized by quantile normalization before statistical analysis. 47 cells (22 from patient 1, 2 from patient 2 and 23 from patient 3) that showed low alignment to exonic regions (<30% of reads), perhaps due to contamination with genomic DNA, were excluded from analysis (Supplementary Figure 2). Mean alignment to exonic regions for remaining 395 cells was 55.3% (34.4–92.6%). Similar to previous studies^7–11^, we observed several cells to be bi-hormonal - expressing high levels of two hormones from *INS*, *GCG* or *SST*. Most bi-hormonal islet single-cells were previously shown to be false positives^7–10^ and it has been reported that the C1 may capture doublet cells (Fluidigm white-paper). We thus evaluated *INS*, *GCG* and *SST* transcripts expression individually in each cell and excluded 101 cells with >10% expression of secondary hormone compared to the primary hormone expressed.

### Identification of islet cell-types

Hierarchical clustering of 294 cells that passed QC procedures were performed using Hierarchical Cluster Viewer (6.5) on Partek Genomics Suite (ver6.6). Hierarchical clustering of cells were initially performed using all transcripts with RPKM values ≥500 in ≥2% of cells (Supplementary Figure 3) and subsequently, to distinctly separate out various islet cell-types, using expressed transcripts from pancreatic cell lineage genes^12^ (Supplementary Figure 4). These clustering analyses further identified 18 possible doublet cells (Supplementary Figure 4) that were excluded before statistical analyses.

We excluded 47 exocrine cells (with high *REG1A* expression) and 18 doublet cells and performed additional hierarchical clustering and PCA analyses based on *INS*, *GCG* and *SST* transcript expression levels (Supplementary Figure 5). These analyses revealed that the 118 *GCG* expressing cells (33 from patient 1, 15 from patient 2 and 70 from patient 3), 105 *INS* expressing cells (32 from patient 1, 33 from patient 2 and 40 from patient 3) and 6 *SST* expressing cells (1 from patient 2 and 5 from patient 3) were predominantly mono-hormonal. Additional hierarchical clustering to identify possible within subtype groups of cells were performed using 118 *GCG* expressing cells (Supplementary Figure 6) and 105 *INS* expressing cells (Supplementary Figure 7).

### Statistical analysis

ANOVA analyses were performed to identify transcripts that were differentially expressed between *INS* expressing cells and high *GCG* expressing cells. To identify gene expression signatures among the *INS* and *GCG* expressing cells in our study, we utilized cells from patient 1 and 2 as the discovery stage (65 high *INS* expressing cells and 48 high *GCG* expressing cells) and cells from patient 3 for validation 40 high *INS* expressing cells and 70 high *GCG* expressing cells). ANOVA analyses were restricted to more highly expressed [582 transcripts (412 genes) with RPKM >50 in at least 25% of cells). P-values were adjusted by Bonferroni correction for the number of tests performed (582 tests) and significant associations were defined as those with an adjusted p-value < 0.05 and fold-change >or <1.5.

All uniquely expressed genes in α- or β-cells in non-diabetic human and mouse single islet cell studies^2–6^ were selected for validation among our East-Asian islet cells. To improve power to validate these recent findings, all α- and β-cells from patients 1, 2 and 3 in our study were utilized (118 α-cells and 105 β-cells). ANOVA analyses, as indicated above, were performed to identify transcripts that were differentially expressed between our East-Asian α- and β-cells. P-values were adjusted by Bonferroni correction for the number of tests performed and significant associations were defined as those with an adjusted p-value < 0.05 and fold-change >or <1.5.

Ingenuity pathway analysis (IPA ver1-07) was used to evaluate for potential upstream regulators and over-representation in canonical pathways for identified genes in our study.

### Immunohistochemistry validation

Human pancreatic samples were fixed in 4% paraformaldehyde followed by 30% sucrose overnight before embedding in tissue freezing medium (Leica, Germany). Sections were prepared on Superfrost Plus (Fisher Scientific, USA) at 10 um thickness. Thereafter slides were blocked for 1 hr at room temperature followed by incubation with primary antibodies (Guinea Pig Anti-insulin (Dako, Denmark), Mouse Anti-Glucagon (Sigma Aldrich, USA), Rabbit Anti-DNAJC3 (Abcam, USA), Rabbit Anti-PFKFB2 (Abcam, USA), and Mouse Anti-UCHL1 (Sigma Aldrich, USA) overnight in 1X TBS with 1% BSA at 4 °C. Slides were washed in 1X TBS with 0.025% Triton X-100 and incubated with secondary antibodies Alexa 647 Anti-Guinea Pig (Life Tech, USA), Alexa 568 Anti-Mouse (LifeTech, USA), Alexa 488 Anti-Rabbit (LifeTech, USA), Alexa 488 Anti-Mouse (Life tech, USA), DAPI (LifeTech, USA) at room temperature for 1 hr. Following incubation and washing, slides were mounted with Vectashield mounting media (Vectalabs, USA). Images were taken using a Zeiss LSM800 and analysed with ZEN Blue® software.

## Results

### Islet cell-types

Hierarchical clustering of human islet cells using all transcripts with RPKM values ≥500 in ≥2% of cells [252 transcripts (146 genes)] revealed three main clusters based on known islet cell transcripts at *GCG*, *INS* and *REG1A*, indicating that cells captured were mainly pancreatic endocrine (α- and β-cells) and exocrine cells (Supplementary Figure 3). Cells were subsequently clustered using highly expressed transcripts (RPKM values ≥500 in ≥2% of cells) from pancreatic cell lineage genes^12^ together with *REG1A* to distinctly separate out islet cell-types (Supplementary Figure 4). These analyses further identified and excluded 18 doublet cells due to dual expression of transcripts from *REG1A*, *GCG*, *INS* and *SST*. Remaining 276 cells were assigned as 47 *REG1A* expressing exocrine cells, 118 *GCG* expressing α-cells, 105 *INS* expressing β-cells and 6 *SST* expressing δ-cells (Supplementary Figures 4 and 5). Hierarchical clustering of assigned α-cells and β-cells did not reveal distinct sub-groups of cell-types (Supplementary Figures 6 and 7).

46,390 transcripts from 17,287 genes were detected among the 276 cells that passed QC filters. On average 14,796 transcripts (8,450–19,547) and 5,638 genes (3,136–7,601) were expressed per cell (Supplementary Figure 8). Similar to previous reports^5, 6^, a large proportion of transcripts were detected at relatively low levels – approximately 25% of transcripts with total RPKM <5.

### Differentially expressed transcripts among β-cells and α-cells

To identify β- and α-cell gene expression patterns among our data, we utilized cells from patient 1 and 2 in a discovery stage. ANOVA analysis was performed among 65 β-cells and 48 α-cells in the discovery stage. Identified transcripts were subsequently validated in 40 β-cells and 70 α-cells from patient 3. We identified 12 transcripts (11 genes - *INS*, *HADH*, *SEC11C*, *PFKB2*, *MAP1B*, *SCD*, *RNR2*, *OLMALINC*, *DNAJC3*, *UCHL1* and *G6PC2*) to be highly expressed in β-cells and 17 transcripts (17 genes - *GCG*, *TTR*, *ALDH1A1*, *CLU*, *TMEM176B*, *GC*, *BEX3*, *HIGD1A*, *ELL2*, *MYL6*, *CYSTM1*, *H3F3B*, *LDHA*, *NAA20*, *FABP5*, *STMN1*, *RBX1*) to be highly expressed among α-cells (Table 1). Ingenuity pathway analysis (IPA ver1-07) of replicating genes indicated involvement in multiple nuclear receptor and AMPK signaling pathways (Supplementary Figure 9) and revealed FOXA2, MAFA, HNF1A, NKX6-2 as top upstream transcriptional regulators (Supplementary Table 2). Differentially expressed β and α-cell genes with increased level of stringency, where highly expressed transcripts were cut-off based on proportion of cells in the smallest islet cell endocrine group in the discovery stage [232 transcripts (314 genes) with RPKM >50 in at least 42% of cells] and replication stage [202 transcripts (298 genes) with RPKM >50 in at least 47% of cells] is also provided in Supplementary Table 3.

**Table 1:** Significant transcripts and genes from ANOVA analysis of (1) β-cells compared to α-cells and (2) α-cells compared to β-cells in our study.

Gene	Transcript	Discovery stage (65 β-cells vs 48 α-cells)		Validation stage (40 β-cells vs 70 α-cells)	
		Adj p-value	Fold Change	Adj p-value	Fold Change
***ANOVA β-cells compared to α-cells***					
*INS*	NM_000207	1.74 × 10^−35^	343.66	2.65 × 10^−44^	449.61
*INS*	NM_001185097	2.59 × 10^−11^	77.18	4.26 × 10^−15^	351.20
*HADH*	NM_005327	6.74 × 10^−08^	31.08	1.25 × 10^−11^	30.08
*SEC11C*	NM_033280	2.35 × 10^−07^	3.20	1.15 × 10^−02^	1.78
*PFKFB2*	NM_006212	3.23 × 10^−07^	8.96	8.19 × 10^−10^	7.31
*MAP1B*	NM_001324255	1.68 × 10^−05^	2.40	1.00 × 10^−03^	2.14
*SCD*	NM_005063	2.07 × 10^−04^	3.53	6.25 × 10^−04^	2.64
*RNR2*	NR_137295	4.92 × 10^−04^	2.35	8.50 × 10^−06^	2.46
*OLMALINC*	NR_026762	3.24 × 10^−03^	5.65	3.43 × 10^−11^	6.98
*DNAJC3*	NM_006260	5.12 × 10^−03^	2.12	4.12 × 10^−03^	1.90
*UCHL1*	NM_004181	6.18 × 10^−03^	2.99	2.33 × 10^−02^	2.07
*G6PC2*	NM_001081686	1.14 × 10^−02^	7.14	2.18 × 10^−07^	12.61
*SPP1*	NM_001040058	1.83 × 10^−02^	100.12	1	1.88
*SPP1*	NM_000582	3.60 × 10^−02^	86.44	1	−1282.90
***ANOVA α-cells compared to β-cells***					
*GCG*	NM_002054	3.39 × 10^−50^	200.93	6.04 × 10^−44^	320.77
*TTR*	NM_000371	9.66 × 10^−21^	20.38	7.86 × 10^−18^	22.12
*ALDH1A1*	NM_000689	3.50 × 10^−06^	7.13	4.16 × 10^−15^	14.86
*CLU*	NM_001831	5.59 × 10^−11^	11.84	4.31 × 10^−15^	6.59
*TMEM176B*	NM_001101312	2.41 × 10^−09^	8.47	2.67 × 10^−13^	7.36
*GC*	NM_001204307	3.96 × 10^−16^	51.21	4.06 × 10^−13^	190.04
*BEX3*	NM_206917	3.52 × 10^−02^	2.29	2.59 × 10^−08^	3.77
*HIGD1A*	NM_014056	1.70 × 10^−07^	3.76	1.24 × 10^−07^	3.44
*ELL2*	NM_012081	3.06 × 10^−09^	3.36	3.73 × 10^−07^	7.38
*MYL6*	NM_079423	4.40 × 10^−07^	3.75	3.35 × 10^−06^	4.28
*CYSTM1*	NM_032412	1.19 × 10^−02^	1.85	5.82 × 10^−06^	2.41
*H3F3B*	NM_005324	7.78 × 10^−05^	2.09	7.08 × 10^−06^	2.42
*LDHA*	NM_005566	2.92 × 10^−03^	4.88	4.03 × 10^−04^	9.27
*NAA20*	NM_016100	1.95 × 10^−02^	1.92	4.67 × 10^−04^	2.32
*FABP5*	NM_001444	1.90 × 10^−05^	8.79	6.04 × 10^−04^	5.40
*STMN1*	NM_005563	1.24 × 10^−02^	3.59	2.02 × 10^−03^	3.55
*RBX1*	NM_014248	4.18 × 10^−02^	2.29	5.82 × 10^−03^	1.99
*CD63*	NM_001780	2.57 × 10^−02^	1.99	5.43 × 10^−02^	1.45
*COX17*	NM_005694	3.59 × 10^−05^	1.81	6.05 × 10^−02^	1.58
*SERPINA1*	NM_000295	8.99 × 10^−09^	25.42	8.23 × 10^−02^	15.47
*SH3BGRL3*	NM_031286	1.78 × 10^−04^	4.33	0.200	1.83
*MARCKS*	NM_002356	4.02 × 10^−02^	1.78	0.659	1.37
*BRK1*	NM_018462	3.02 × 10^−02^	2.02	0.992	1.39
*YWHAE*	NM_006761	2.04 × 10^−02^	1.51	1	1.05
*PPIA*	NM_021130	6.23 × 10^−04^	2.11	1	1.06

Cells from patient 1 and 2 used in discovery stage and cells from patient 3 used for validation. Discovery stage p-value adjusted for 582 transcripts tested (RPKM >50 in at least 25% of cells). Validation stage p-value adjusted for 14 and 25 transcripts tested, respectively.

**Figure 1:**
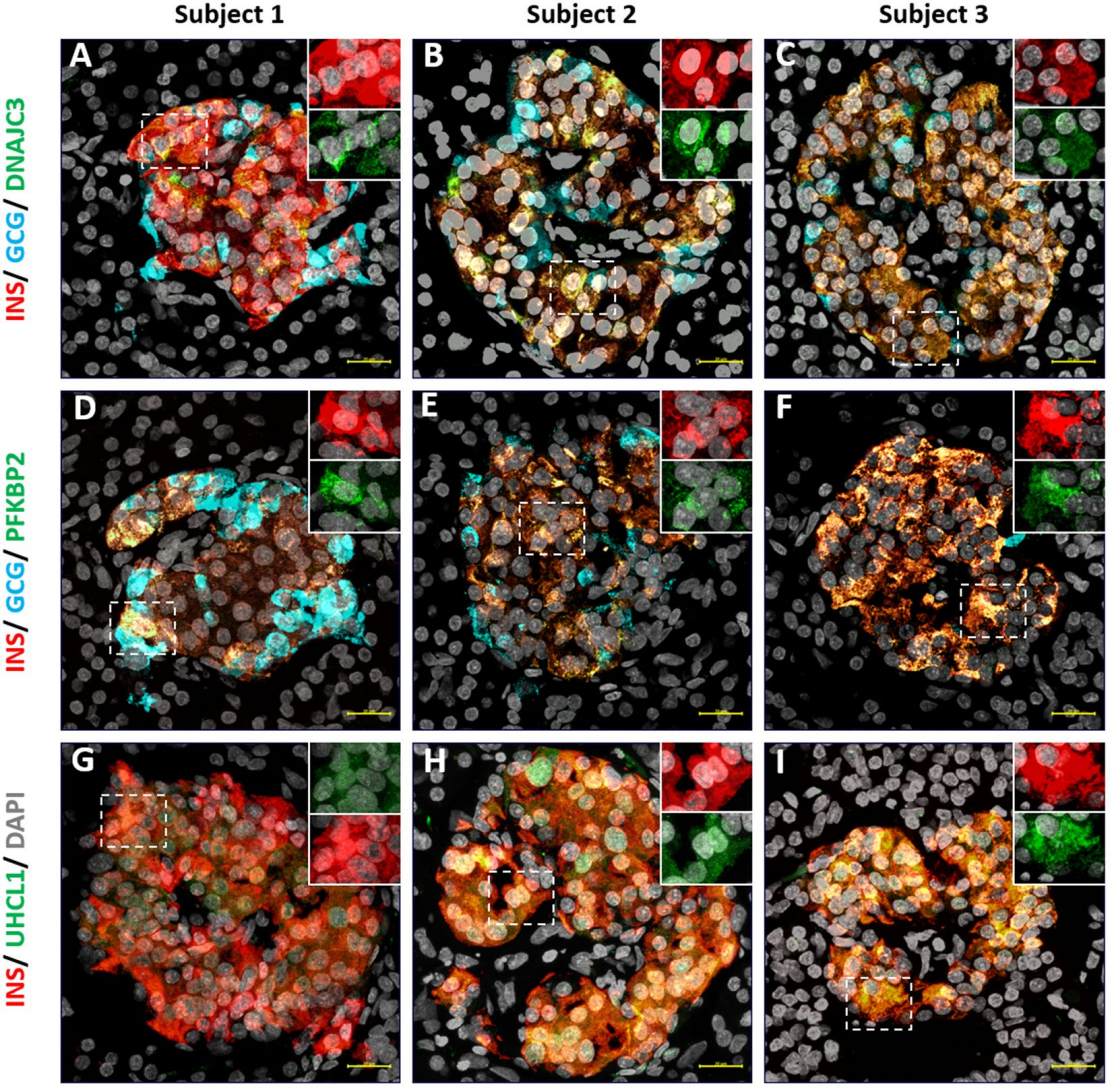
Representative immunofluorescence staining showing co-localization of transcripts with INS + cells and not GCG + cells from pancreas of three different subjects. Subject 1 (**A**,**D** and **G**), subject 2 (**B**,**E** and **H**) and subject 3 (**C**,**F** and **I**). (**A**,**B** and **C**) shows DNAJC3 (green), INS (red), GCG (blue) and nucleus (grey) stains. (**D**,**E** and **F**) shows PFKB2 (green), INS (red), GCG (blue) and nucleus (grey) stains. (**G**,**H** and **I**) shows UCHL1 (green), INS (red) and nucleus (grey) stains. Cell co-expressing INS and DNAJC or PFKB2 or UCHL1 are represented as yellow. Inserts show cells expressing insulin (red) and the respective gene of interest (green) at higher magnification and at individual channels. Scale bar represents 20 µm.

For three hits (DNAJC3, UCHL1 and PFKB2) that showed higher expression levels in our β-cells (as compared to α-cells) we further performed immunohistochemistry validations on islet sections of the three subjects used in the study. All three hits showed strong co-expression in cells producing INS and DNAJC3 and PFKB2 did not co-localize with GCG (Fig. 1, Supplementary Figures 10–12). UHCL1 with GCG co-stains were not carried out because of primary antibody incompatibility.

### *In silico* replication of rodent and human α- and β-cells gene expression signatures

We next evaluated, *in silico*, unique gene expression signatures of non-diabetic mice and human α- and β-cells^7–11^ among our East-Asian scRNA-seq results utilizing cells from all three subjects (118 α-cells and 105 β-cells). ANOVA analyses, using 624 transcripts (584 genes) with RPKM >50 in at least 25% of cells, were performed to identify transcripts that were differentially expressed between our East-Asian α- and β-cells.

We replicated five upregulated mouse α-cell genes (*GCG*, *TTR*, *HIGD1A*, *GPX3* and *PDK4*, adjusted P-value between 4.96 × 10^−98^ and 1.36 × 10^−5^) and five upregulated mouse β-cell genes (*INS*, *HADH*, *G6PC2*, *RRAGD* and *IAPP*, adjusted P-value between 3.93 × 10^−82^ and 3.25 × 10^−5^) among our scRNA-seq data (Supplementary Table 4). *FAM46A* that was reported to be upregulated in mouse β-cells^7^ however showed higher expression in our human α-cells (adjusted P-value = 4.21 × 10^−4^).

Validation of human non-diabetic islet gene expression^8–11^ showed concordantly high expression of transcripts in sixteen genes among our α-cells (*GCG*, *TTR*, *GC*, *TMEM176B*, *HIGD1A*, *GPX3*, *TM4SF4*, *FABP5*, *LDHA*, *NAA20*, *CRYBA2*, *SERPINA1*, *PDK4*, *HLA-E*, *PALLD* and *C10orf10*, adjusted P-value between 2.28 × 10^−97^ and 0.01) and 14 genes among our β-cells (*INS*, *HADH*, *PFKB2*, *G6PC2*, *RPL3*, *EIF4A2*, *RRAGD*, *HNRNPC*, *IAPP*, *CANX*, *FAM162A*, *ACAT1*, *TVP23B* and *SERINC1*, adjusted P-value between 2.81 × 10^−81^ and 0.04) (Tables 2 and 3). Association results of all 624 transcripts tested are indicated in Supplementary Table 5. The validated common (across European and East-Asian ancestry) α-cells genes were over-represented in nuclear receptor signaling as well as the acute phase response signaling pathway and the common β-cells genes were implicated in six pathways (Supplementary Figure 13).

**Table 2:** *In silico* replication of unique α-cells upregulated genes reported in recent human non-diabetic islet single-cell studies^8–11^. Adjusted P-value corrected for 23 transcripts tested.

Gene	Transcript	P-value	Adj p-value	Fold Change
GCG	NM_002054	9.92 × 10^−99^	2.28 × 10^−97^	223.04
TTR	NM_000371	2.10 × 10^−42^	4.83 × 10^−41^	20.22
GC	NM_001204307	1.48 × 10^−30^	3.40 × 10^−29^	47.89
TMEM176B	NM_001101312	1.14 × 10^−25^	2.62 × 10^−24^	8.49
HIGD1A	NM_014056	3.39 × 10^−16^	7.79 × 10^−15^	3.21
GPX3	NM_002084	3.32 × 10^−13^	7.65 × 10^−12^	3.14
TM4SF4	NM_004617	5.94 × 10^−13^	1.37 × 10^−11^	46.74
FABP5	NM_001444	7.48 × 10^−12^	1.72 × 10^−10^	6.70
LDHA	NM_005566	7.23 × 10^−10^	1.66 × 10^−08^	5.59
NAA20	NM_016100	3.37 × 10^−09^	7.76 × 10^−08^	2.04
CRYBA2	NM_057094	6.86 × 10^−09^	1.58 × 10^−07^	30.65
SERPINA1	NM_000295	4.83 × 10^−08^	1.11 × 10^−06^	16.31
PDK4	NM_002612	2.72 × 10^−06^	6.26 × 10^−05^	6.97
HLA-E	NM_005516	7.51 × 10^−06^	1.73 × 10^−04^	2.66
PALLD	NM_001166110	1.98 × 10^−04^	4.55 × 10^−03^	2.06
C10orf10	NM_007021	4.40 × 10^−04^	0.0101	2.39
ITGB1	NM_133376	8.13 × 10^−03^	0.1870	1.58
SSR4	NM_006280	0.0149	0.3433	1.23
PRDX4	NM_006406	0.0763	1	1.30
FKBP2	NM_004470	0.0850	1	1.39
TUBA1B	NM_006082	0.0969	1	1.16
TAPBP	NM_172209	0.4096	1	1.15
PEMT	NM_148173	0.4437	1	1.12

**Table 3:** *In silico* replication of unique β-cells upregulated genes reported in recent human non-diabetic islet single-cell studies^8–11^. Adjusted P-value corrected for 50 transcripts tested.

Gene	Transcript	p-value	Adj p-value	Fold Change
INS	NM_000207	5.62 × 10^−83^	2.81 × 10^−81^	415.58
INS	NM_001185097	3.24 × 10^−30^	1.62 × 10^−28^	132.44
HADH	NM_005327	2.26 × 10^−21^	1.13 × 10^−19^	31.35
PFKFB2	NM_006212	3.93 × 10^−19^	1.96 × 10^−17^	10.90
G6PC2	NM_001081686	3.09 × 10^−12^	1.54 × 10^−10^	9.95
RPL3	NM_000967	8.93 × 10^−09^	4.46 × 10^−07^	1.69
EIF4A2	NM_001967	1.84 × 10^−08^	9.21 × 10^−07^	1.68
RRAGD	NM_021244	1.26 × 10^−07^	6.29 × 10^−06^	2.92
HNRNPC	NM_004500	5.52 × 10^−07^	2.76 × 10^−05^	1.77
IAPP	NM_000415	4.64 × 10^−06^	2.32 × 10^−04^	317.52
CANX	NM_001024649	1.02 × 10^−05^	5.09 × 10^−04^	1.85
FAM162A	NM_014367	4.78 × 10^−05^	2.39 × 10^−03^	2.08
ACAT1	NM_000019	1.40 × 10^−04^	7.00 × 10^−03^	2.16
TVP23B	NM_001316920	2.04 × 10^−04^	0.0102	1.77
SERINC1	NM_020755	8.55 × 10^−04^	0.0428	1.69
ATP6V1A	NM_001690	1.15 × 10^−03^	0.0576	1.59
CCNI	NM_006835	1.72 × 10^−03^	0.0860	1.69
NDUFA9	NM_005002	3.51 × 10^−03^	0.1753	−1.73
SLC30A8	NM_173851	0.0120	0.5996	1.55
NACA	NM_001113202	0.0163	0.8151	1.32
TMEM60	NM_032936	0.0249	1	1.89
TMEM33	NM_018126	0.0256	1	1.28
SDHB	NM_003000	0.0362	1	−1.43
COMMD3	NM_012071	0.0416	1	1.50
METTL5	NM_001293187	0.0542	1	1.43
SDCBP	NM_005625	0.0774	1	1.36
SDCBP	NM_001007069	0.0891	1	1.42
IRF2BP2	NM_182972	0.1023	1	1.31
SLC39A9	NM_001252151	0.1069	1	1.38
IRF2BP2	NM_001077397	0.1124	1	1.31
SLC39A9	NM_001252152	0.1167	1	1.38
HSPA8	NM_006597	0.1405	1	1.27
TMED2	NM_006815	0.1610	1	1.23
SCGN	NM_006998	0.1626	1	1.21
HSP90AB1	NM_007355	0.2462	1	1.10
JKAMP	NM_016475	0.4063	1	1.20
SLC30A8	NM_001172814	0.4398	1	1.15
RAB1A	NM_004161	0.4521	1	−1.14
PSMD7	NM_002811	0.4784	1	1.11
CSDE1	NM_007158	0.4910	1	1.12
TXLNA	NM_175852	0.5687	1	1.10
ARF6	NM_001663	0.5739	1	−1.15
ANAPC13	NM_015391	0.6284	1	−1.07
EIF3M	NM_006360	0.6714	1	−1.07
PEBP1	NM_002567	0.7090	1	1.05
ANXA2	NM_004039	0.7236	1	1.15
MRPS33	NM_053035	0.7240	1	−1.06
CACUL1	NM_153810	0.7407	1	1.05
NDUFB5	NM_002492	0.7877	1	1.05
MAPRE1	NM_012325	0.8704	1	−1.02

Nine α-cell genes (*ALDHA1, CLU, BEX3, ELL2, MYL6, CYSTM1, H3F3B, STMN1 and RBX1*) and seven β-cell genes (*OLMALINC, RNR2, SCD, MAP1B, DNAJC3, SEC11C and UCHL1)* were significantly expressed in our East-Asian data (Table 1) but not reported as unique non-diabetic upregulated human α- or β-cell genes^8–11^. IPA analysis of these genes revealed a role for protein ubiquitination pathway among East-Asian β-cells (Supplementary Figures 14 and 15), while no pathway remained significant among the discordant α-cell genes after multiple test corrections.

## Discussion

We utilized Fluidigm C1™ to capture single islet cells from non-diabetic East-Asian subjects and utilized these for RNA-seq analyses. Reassuringly the main genes expressed in our scRNA-seq data of human islets were *INS* and *GCG*, indicating that predominantly endocrine cells were captured. Besides these determinant gene classifiers, our data corroborated the role of several well-characterized as well as more novel α- and β-cell genes identified very recently^7–11^. Pathway-based analyses were used to evaluate the role of genes in aggregate and substantiated multiple known pathways such as nuclear receptor activations and AMPK signaling, in addition to more novel ones, such as the EIF2 signaling in β-cells^7–11^.

We report on specific mouse-human species difference in islet cell transcriptomic patterns, where mouse β-cell gene, *FAM46A*, showed higher expression in our human α-cells instead. Such species differences in expression patterns for other genes, such as *GC* and *DLK1*, have been identified from previous studies^9, 10^ and may reflect functional differences at the species level^13^.

Our East-Asian scRNA-seq data enabled for an ethnic-specific evaluation of transcriptomic signatures with recently published data. Besides the nuclear receptor activation pathways, identification of acute phase response signaling as a common pathway involved in α-cells reinforces the view that α-cells may be better protected, as compared to β-cells, against microorganism infections^14^. Similarly identification of glutaryl-coA and tryptophan degradations may indicate on the importance of adiposity or cholesterol levels in influencing β-cell functions^15, 16^.

Interestingly, we identified the protein ubiquitination pathway that included *DNAJC3* and *UCHL1* genes, as a pathway in East-Asian β-cells. These genes have been associated with T2D and perhaps indicate the vulnerability of pancreatic β-cells for protein misfolding, increased ER stress and proteotoxicity, hallmarks for islet cell defects in T2D^17–20^. Intriguingly, comparing our β-cell gene signatures in non-diabetic East-Asian individuals to genes highly expressed in β-cells of T2D subjects^11^ also revealed transcript at *HSP90B1* that is involved in ER stress (data not shown). Our comparative analyses therefore indicate that pancreatic β-cells in non-diabetic East-Asians may already be predisposed to increased ER stress and subsequent cell death. Nevertheless, given that present human islet single-cell studies are still presently modest in the number of cells analyzed, whether this protein ubiquitination pathways render β-cells more susceptible to cell death and if these result in subsequent development of T2D disease risks, warrants further validations in large-scale East-Asian islet single-cells, including subjects at various disease states (i.e. pre-diabetes and type 2 diabetes) and specific functional evaluations.

Our study has limitations such as the high number of doublet cells and may indicate experimental contaminations. This however, is a common feature among islet single-cell studies that resulted in the exclusion of about 30% of captured cells^7–10^. We believe that our stringent QC procedures that excluded a similar proportion of cells, would have removed most contaminated data from analyses. Another limitation was that we had restricted our analyses to highly expressed transcripts to identify differential ones between α- and β-cells. This may have resulted in a reduced number of mouse and human islet transcripts tested during *in silico* validations. However, due to the relatively modest number of cells analyzed, we have reduced power to identify rare transcripts as well as to identify sub-groups of cells.

In conclusion, we present for the first time scRNA-seq data from non-diabetic East-Asian islets and corroborated multiple genes and pathways that may be commonly involved in α- and β-cells across ancestry groups. At the same time we identify signatures that may play a more prominent role in East-Asians, such as those involved in protein ubiquitination, and these require subsequent evaluations to fully appreciate their roles in diabetes etiology.

## Electronic supplementary material

Supplementary Tables and Figures
