# Supplementary material for: Single-cell transcriptomics of East-Asian pancreatic islets cells
Source: Sci Rep. 2017 Jul 10;7:5024. doi: 10.1038/s41598-017-05266-4 (PMC5504042; doi:10.1038/s41598-017-05266-4)

Single-cell transcriptomics of East-Asian pancreatic islets cells

Rajkumar Dorajoo, Yusuf Ali, Vanessa SY Tay, Jonathan Kang , Sudhagar Samydurai, Jianjun Liu and Bernhard O. Boehm

Supplementary Tables

Supplementary Table 1: Details of human islets utilized in study. Glucose Stimulated Insulin Secretion (GSIS) data is shown as area under the curve, which reflects amount of insulin secreted per 10 minutes.

Subject	Age	Ethnicity	Sex	HbA1c (%)	Days in Culture	Islet-Glucose Stimulated Insulin Secretion		Cells captured on Fluidigm C1		
						Low Glucose	High Glucose	Total cells	β-cells passed QC	α-cells passed QC
1	65	Chinese	Male	5.9	3	48.2	299.9	138	32	33
2	49	Chinese	Female	5.8	3	64.9	1187.3	84	33	15
3	49	Chinese	Female	5.1	3	87.9	732.2	226	40	70

Supplementary table 2: Identification of top 5 upstream transcriptional regulators from significant gene showing high expression in β-cells and α-cells.

Upstream transcription factors	P-value	Target molecules in dataset
14 genes highly expressed in β-cells		
FOXA2	9.04E-07	*G6PC2,HADH,MAP1B,SCD*
MAFA	9.10E-06	*G6PC2,INS*
XBP1	7.59E-05	*DNAJC3,INS,SEC11C*
MAF	2.17E-04	*G6PC2,INS*
HNF1A	7.23E-04	*G6PC2,INS,PFKFB2*
20 genes highly expressed in α-cells		
FOXM1	7.78E-05	*GCG,LDHA,STMN1*
H2AFX	1.15E-04	*CLU,MYL6*
FOXA2	3.44E-04	*FABP5,GCG,TTR*
MYC	7.18E-04	*CLU,FABP5,H3F3A/H3F3B,LDHA,STMN1*
NKX6-2	7.30E-04	*GCG*

Supplementary table 3: Significant transcripts and genes from ANOVA analysis of 1) β-cells compared to α-cells and 2) α-cells compared to β-cells in our study. Cells from patient 1 and 2 used in discovery stage and cells from patient 3 used for validation. Highly expressed transcripts were cut-off based on proportion of cells in the smallest islet cell endocrine group in the discovery stage [232 transcripts (314 genes) with RPKM > 50 in at least 42% of cells] and replication stage [202 transcripts (298 genes) with RPKM > 50 in at least 47% of cells]. Discovery stage p-value adjusted for 232 transcripts tested. Validation stage p-value adjusted for 8 and 16 transcripts tested, respectively.

		Discovery stage (65 β-cells vs 48 α-cells)		Validation stage (40 β-cells vs 70 α-cells)	
**Gene**	**Transcript**	**Adj p-value**	**Fold Change**	**Adj p-value**	**Fold Change**
ANOVA β-cells compared to α-cells					
INS	NM_000207	6.94E-36	343.6	1.81E-47	449.61
INS	NM_001185097	1.03E-11	77.18	7.85E-16	351.20
HADH	NM_005327	2.69E-08	31.08	1.13E-10	30.08
PFKFB2	NM_006212	1.29E-07	8.96	7.37E-09	7.31
MAP1B	NM_001324255	6.70E-06	2.40	3.02E-05	2.14
RNR2	NR_137295	1.96E-04	2.35	1.99E-04	2.46
OLMALINC	NR_026762	1.29E-03	5.65	1.52E-12	6.98
DNAJC3	NM_006260	2.04E-03	2.12	5.30E-04	1.92
ANOVA α-cells compared to β-cells					
GCG	NM_002054	1.35E-50	200.93	5.15E-44	320.77
TTR	NM_000371	3.85E-21	20.38	4.64E-18	22.12
ALDH1A1	NM_000689	1.39E-06	7.13	2.05E-15	14.86
CLU	NM_001831	2.23E-11	11.84	5.05E-14	6.59
TMEM176B	NM_001101312	9.58E-10	8.47	7.30E-13	7.36
BEX3	NM_206917	1.40E-02	2.29	1.10E-07	3.77
HIGD1A	NM_014056	6.80E-08	3.76	1.20E-06	3.44
ELL2	NM_012081	1.22E-09	3.36	4.16E-06	7.38
MYL6	NM_079423	1.75E-07	3.75	6.24E-05	4.28
H3F3B	NM_005324	3.11E-05	2.09	5.63E-06	2.42
LDHA	NM_005566	1.16E-03	4.88	6.56E-04	9.27
NAA20	NM_016100	7.75E-03	1.92	1.05E-03	2.32
FABP5	NM_001444	7.56E-06	8.79	5.15E-04	5.43
SH3BGRL3	NM_031286	7.08E-05	4.33	1	1.83
YWHAE	NM_006761	8.12E-03	1.51	1	1.05
PPIA	NM_021130	2.48E-04	2.11	1	1.06

Supplementary table 4: *In silico* replication of unique α-cells upregulated genes and β-cells upregulated genes reported in recent mouse islet single-cell studies [2]. Adjusted P-value corrected for 5 and 7 transcripts tested, respectively.

Gene	Transcript	p-value	Adj p-value	Fold Change
**Validation of highly expressed mouse α-cell genes**				
GCG	NM_002054	9.92E-99	4.96E-98	223.04
TTR	NM_000371	2.10E-42	1.05E-41	20.22
HIGD1A	NM_014056	3.39E-16	1.69E-15	3.22
GPX3	NM_002084	3.32E-13	1.66E-12	3.15
PDK4	NM_002612	2.72E-06	1.36E-05	6.97
Validation of highly expressed mouse β-cell genes				
INS	NM_000207	5.62E-83	3.93E-82	415.58
INS	NM_001185097	3.24E-30	2.26E-29	132.44
HADH	NM_005327	2.26E-21	1.58E-20	31.35
G6PC2	NM_001081686	3.09E-12	2.16E-11	9.95
RRAGD	NM_021244	1.26E-07	8.81E-07	2.93
IAPP	NM_000415	4.64E-06	3.25E-05	317.52
FAM46A	NM_017633	6.01E-05	4.21E-04	-2.35

Supplementary table 5: Association details of all 624 transcripts with RPKM values > 50 in 25% of cells in ANOVA analysis of 105 β-cells compared 118 α-cells from all 3 non-T2D East-Asian subjects.

Gene Symbol	Transcript	p-value	Fold Change
GCG	NM_002054	9.92E-99	-223.044
INS	NM_000207	5.62E-83	415.576
TTR	NM_000371	2.10E-42	-20.222
GC	NM_001204307	1.48E-30	-47.890
INS	NM_001185097	3.24E-30	132.443
CLU	NM_001831	1.26E-29	-8.729
TMEM176B	NM_001101312	1.14E-25	-8.492
ALDH1A1	NM_000689	4.81E-25	-10.942
HADH	NM_005327	2.26E-21	31.354
PFKFB2	NM_006212	3.93E-19	10.902
HIGD1A	NM_014056	3.39E-16	-3.217
OLMALINC	NR_026762	3.62E-16	6.532
MAP1B	NM_001324255	5.64E-15	2.658
ELL2	NM_012081	1.79E-14	-3.270
H3F3B	NM_005324	3.92E-14	-2.251
BEX3	NM_206917	4.07E-14	-3.279
MYL6	NM_079423	6.07E-14	-3.479
GPX3	NM_002084	3.32E-13	-3.148
TM4SF4	NM_004617	5.94E-13	-46.744
CYSTM1	NM_032412	1.36E-12	-2.186
SEC11C	NM_033280	2.12E-12	2.367
G6PC2	NM_001081686	3.09E-12	9.950
RNR2	NR_137295	3.68E-12	2.480
FABP5	NM_001444	7.48E-12	-6.704
SCD	NM_005063	1.45E-11	3.091
ERO1B	NM_019891	4.49E-10	3.052
LDHA	NM_005566	7.23E-10	-5.592
CHGB	NM_001819	7.97E-10	-2.689
RPL5	NM_000969	1.68E-09	1.763
UCHL1	NM_004181	2.42E-09	2.640
NAA20	NM_016100	3.37E-09	-2.042
STMN1	NM_005563	4.24E-09	-3.527
RPS6	NM_001010	4.67E-09	1.445
CRYBA2	NM_057094	6.86E-09	-30.647
RPS3A	NM_001006	7.93E-09	1.515
RPL3	NM_000967	8.93E-09	1.696
EIF4A2	NM_001967	1.84E-08	1.687
CD63	NM_001780	2.38E-08	-1.821

SCG2	NM_003469	2.84E-08	-1.963
RPL34	NM_001319236	4.30E-08	1.658
SERPINA1	NM_000295	4.83E-08	-16.314
ROMO1	NM_080748	1.09E-07	-2.150
RRAGD	NM_021244	1.26E-07	2.929
SH3BGRL3	NM_031286	1.65E-07	-2.562
NDUFA13	NM_015965	1.72E-07	-2.016
HMGN2	NM_005517	3.01E-07	-1.698
COX8A	NM_004074	3.11E-07	-1.831
RPS4X	NM_001007	3.23E-07	1.661
HNRNPC	NM_004500	5.52E-07	1.771
PCBD1	NM_000281	6.17E-07	-2.238
TPT1	NM_003295	1.22E-06	1.515
RPS13	NM_001017	1.33E-06	1.516
NDUFB2	NM_004546	1.44E-06	-1.593
COX17	NM_005694	1.86E-06	-1.525
STMN1	NM_203399	2.54E-06	-2.758
MALAT1	NR_002819	2.57E-06	1.421
PDK4	NM_002612	2.72E-06	-6.971
CTTN	NM_138565	3.69E-06	-2.123
DNAJC3	NM_006260	4.39E-06	1.845
IAPP	NM_000415	4.64E-06	317.515
HIST1H4C	NM_003542	7.49E-06	1.635
HLA-E	NM_005516	7.51E-06	-2.656
COX6A1	NM_004373	8.01E-06	-1.524
RBX1	NM_014248	9.25E-06	-1.851
CANX	NM_001024649	1.02E-05	1.856
ERGIC3	NM_015966	1.13E-05	-2.730
DNAJB9	NM_012328	1.29E-05	3.037
NDUFB8	NM_005004	1.32E-05	-1.801
UBB	NM_018955	1.42E-05	-1.797
ARHGAP1	NM_004308	1.55E-05	-1.993
RPS3	NM_001256802	1.98E-05	1.416
PARK7	NM_001123377	2.55E-05	-1.573
HNRNPA2B1	NM_002137	2.96E-05	-1.709
MARCKS	NM_002356	3.05E-05	-1.513
MIR7-3HG	NR_027148	3.10E-05	-2.142
ATP5E	NM_006886	3.87E-05	-1.535
UBE2B	NM_003337	4.17E-05	1.925
ALDOA	NM_184043	4.28E-05	1.954
TSC22D1	NM_006022	4.48E-05	2.995

FAM162A	NM_014367	4.78E-05	2.083
SCG3	NM_013243	4.89E-05	1.595
RAB11A	NM_004663	4.98E-05	-1.778
ATP6V1G1	NM_004888	5.39E-05	1.480
FAM46A	NM_017633	6.01E-05	-2.351
CPE	NM_001873	6.87E-05	1.551
RPL7	NM_000971	7.90E-05	1.422
PPP1CB	NM_206876	8.04E-05	1.473
CD164	NM_006016	8.57E-05	-1.943
OSTC	NM_021227	9.19E-05	2.288
BRK1	NM_018462	1.13E-04	-1.568
PJA2	NM_014819	1.22E-04	2.015
SNRPG	NM_003096	1.38E-04	-1.486
HSP90B1	NM_003299	1.39E-04	1.895
ACAT1	NM_000019	1.40E-04	2.165
C4orf3	NM_001001701	1.40E-04	1.764
PSME2	NM_002818	1.56E-04	-1.854
SRSF6	NM_006275	1.58E-04	-1.523
RPL24	NM_000986	1.61E-04	1.371
PALLD	NM_001166110	1.98E-04	-2.066
TVP23B	NM_001316920	2.04E-04	1.775
RPS25	NM_001028	2.09E-04	1.382
RNR1	NR_137294	2.12E-04	2.113
NPC2	NM_006432	2.17E-04	-1.579
RPL14	NM_003973	2.28E-04	1.416
PRDX2	NM_005809	2.39E-04	-1.491
GSTP1	NM_000852	2.41E-04	-1.778
ENO1	NM_001428	2.66E-04	1.569
RPS15	NM_001018	2.78E-04	-1.508
BNIP3L	NM_004331	2.90E-04	1.766
COX7A2	NM_001865	2.94E-04	-1.321
TMEM167A	NM_174909	3.47E-04	2.007
RPS27	NM_001030	3.61E-04	1.290
ATP5B	NM_001686	4.01E-04	-1.563
C10orf10	NM_007021	4.40E-04	2.399
ARF4	NM_001660	4.49E-04	1.665
CLIC1	NM_001287594	5.14E-04	-1.678
RPL31	NM_000993	5.21E-04	1.323
ATP6V0D1	NM_004691	6.57E-04	-2.118
PPP1CB	NM_002709	6.62E-04	1.325
SAT1	NM_002970	6.84E-04	-2.121

PRDX1	NM_181697	7.96E-04	1.692
CALM2	NM_001743	8.01E-04	-1.271
C14orf2	NM_004894	8.08E-04	-1.486
LAPTM4A	NM_014713	8.25E-04	1.397
RTN4	NR_135830	8.45E-04	1.729
SERINC1	NM_020755	8.55E-04	1.692
WSB2	NM_001278558	9.38E-04	1.481
ERGIC2	NM_016570	9.75E-04	1.807
GNAS	NM_001309840	9.76E-04	1.363
RPL4	NM_000968	9.78E-04	1.456
FAU	NM_001997	9.90E-04	-1.468
SSR3	NM_007107	9.99E-04	1.628
HSPE1	NM_002157	1.00E-03	-1.481
RPS12	NM_001016	1.03E-03	1.319
NDUFB1	NM_004545	1.09E-03	-1.483
RTN4	NR_135829	1.09E-03	1.715
ATP6V1A	NM_001690	1.15E-03	1.590
RPL6	NM_000970	1.20E-03	1.398
NDUFA1	NM_004541	1.21E-03	-1.336
OAZ1	NM_004152	1.26E-03	-1.456
CNBP	NM_001127196	1.30E-03	1.674
ERO1A	NM_014584	1.32E-03	2.259
ZFAND6	NM_001242915	1.37E-03	2.308
RHOA	NM_001664	1.44E-03	-1.579
RPL12	NM_000976	1.60E-03	1.314
CCNI	NM_006835	1.72E-03	1.699
EIF3E	NM_001568	1.84E-03	1.574
PFDN5	NM_002624	1.85E-03	1.365
EIF3L	NM_016091	2.03E-03	1.850
ARL6IP1	NM_015161	2.13E-03	1.586
RNASEK	NM_001004333	2.25E-03	-1.355
LINC01420	NR_015367	2.27E-03	-1.698
OTUD6B-AS1	NR_110439	2.41E-03	1.756
RPL13A	NM_012423	2.54E-03	1.509
CNBP	NM_003418	2.62E-03	1.665
ALDOA	NM_184041	2.67E-03	1.532
HINT1	NM_005340	2.78E-03	-1.265
GHITM	NM_014394	2.82E-03	1.450
COA3	NM_001040431	2.96E-03	-1.599
EEF1B2	NM_021121	3.01E-03	1.775
TAF7	NM_005642	3.11E-03	1.622

ENPP2	NR_045555	3.19E-03	-1.962
TMBIM6	NM_003217	3.29E-03	-1.262
CDC42	NM_001791	3.30E-03	-1.368
KRTCAP2	NM_173852	3.45E-03	-1.506
RPS27A	NM_001177413	3.50E-03	1.304
NDUFA9	NM_005002	3.51E-03	-1.783
TLK1	NM_001136555	3.51E-03	1.538
TTC3	NM_001001894	3.56E-03	1.736
MT1X	NM_005952	3.57E-03	3.482
SCGB2A1	NM_002407	3.63E-03	-1.581
SLC25A5	NM_001152	3.71E-03	-1.387
SLC25A3	NM_002635	3.78E-03	-1.746
QPCT	NM_012413	4.16E-03	-1.571
DYNLRB1	NM_014183	4.24E-03	-1.620
COX6B1	NM_001863	4.36E-03	-1.419
RPS14	NM_005617	4.38E-03	1.240
SH3BGRL	NM_003022	4.50E-03	2.012
EEF1G	NM_001404	4.69E-03	1.338
NOP10	NM_018648	4.92E-03	-1.419
PDIA6	NM_005742	4.93E-03	1.501
NEDD8	NM_006156	5.18E-03	-1.389
PSMB3	NM_002795	5.35E-03	-1.429
GTF3C6	NM_138408	5.38E-03	-1.711
GNG5	NM_005274	5.55E-03	-1.659
MORF4L1	NM_001265605	5.84E-03	1.261
PON2	NM_000305	5.89E-03	1.769
RPS15A	NM_001030009	5.94E-03	1.295
SRPRB	NM_021203	6.51E-03	1.499
RPL15	NM_001253383	6.88E-03	1.325
CHURC1	NM_001204063	7.36E-03	1.485
RPL11	NM_000975	7.53E-03	1.316
PGK1	NM_000291	7.53E-03	1.476
ITGB1	NM_133376	8.13E-03	-1.580
ATP5A1	NM_004046	8.22E-03	-1.550
H2AFZ	NM_002106	8.36E-03	1.394
PLP2	NM_002668	8.77E-03	-1.426
UFC1	NM_016406	9.02E-03	-1.459
PTMA	NM_002823	9.39E-03	1.298
RPL30	NM_000989	9.47E-03	1.249
EIF1B	NM_005875	9.94E-03	1.661
DAD1	NM_001344	1.01E-02	1.286

TXNL1	NM_004786	1.01E-02	1.648
FTH1	NM_002032	1.03E-02	1.554
NORAD	NR_027451	1.03E-02	1.374
RPL41	NM_021104	1.06E-02	1.161
BEX1	NM_018476	1.11E-02	-1.330
PSMB6	NM_002798	1.13E-02	-1.649
WTAP	NM_001270531	1.14E-02	1.467
ATP6V1E1	NM_001696	1.17E-02	1.564
SLC30A8	NM_173851	1.20E-02	1.556
CDV3	NM_001282764	1.23E-02	1.619
RPL26	NM_000987	1.26E-02	1.322
PPIA	NM_021130	1.32E-02	-1.358
PSMB1	NM_002793	1.37E-02	1.357
DYNLT3	NM_006520	1.45E-02	1.412
SSR4	NM_006280	1.49E-02	-1.233
USMG5	NM_001206426	1.61E-02	-1.272
NACA	NM_001113202	1.63E-02	1.321
MT2A	NM_005953	1.64E-02	3.492
RACK1	NM_006098	1.67E-02	1.317
ARRDC3	NM_020801	1.77E-02	1.495
PAIP2	NM_016480	1.86E-02	1.448
EEF1B2	NM_001037663	1.87E-02	1.491
MORN2	NM_001145450	1.90E-02	-1.791
PSME1	NM_006263	1.91E-02	-1.409
ATP6AP2	NM_005765	2.03E-02	-1.362
HNRNPDL	NM_031372	2.05E-02	-1.416
ACTG1	NM_001614	2.05E-02	1.504
RPL15	NM_001253382	2.25E-02	1.359
HAX1	NM_006118	2.29E-02	1.482
SRP54	NM_003136	2.36E-02	1.807
RAN	NM_006325	2.41E-02	-1.346
TMEM60	NM_032936	2.49E-02	1.893
TMEM33	NM_018126	2.56E-02	1.283
RPL23	NM_000978	2.58E-02	1.165
EAF1	NM_033083	2.58E-02	1.397
OST4	NM_001134693	2.59E-02	-1.224
RPS5	NM_001009	2.73E-02	1.263
PSMB5	NM_002797	2.77E-02	-1.367
ISL1	NM_002202	2.82E-02	-1.482
YWHAE	NM_006761	2.84E-02	-1.218
NDUFV2	NM_021074	2.89E-02	-1.306

HSP90AA1	NM_005348	2.94E-02	1.563
RGS2	NM_002923	3.11E-02	2.144
POLR2K	NM_005034	3.17E-02	1.373
TMA7	NM_015933	3.19E-02	-1.259
CLK1	NR_027855	3.26E-02	1.670
SARAF	NM_001284239	3.45E-02	1.507
ATP5I	NM_007100	3.47E-02	-1.221
TERF2IP	NM_018975	3.47E-02	1.395
NDUFS4	NM_002495	3.52E-02	-1.466
SDHB	NM_003000	3.62E-02	-1.453
ATP5G1	NM_001002027	3.70E-02	-1.338
GNAS	NM_001309861	3.71E-02	1.261
ARPC2	NM_005731	3.71E-02	-1.368
GAPDH	NM_002046	3.75E-02	-1.256
RPL35A	NM_000996	3.79E-02	1.203
EIF4G2	NM_001042559	3.83E-02	1.353
TMBIM4	NM_016056	3.87E-02	-1.296
S100A11	NM_005620	4.00E-02	-1.669
YWHAG	NM_012479	4.11E-02	1.354
COMMD3	NM_012071	4.16E-02	1.507
ANXA5	NM_001154	4.30E-02	1.428
PDIA3	NM_005313	4.32E-02	-1.378
TAF9	NM_003187	4.38E-02	1.456
RPS20	NM_001023	4.74E-02	1.167
NPM1	NM_002520	4.78E-02	1.190
EIF1	NM_005801	4.80E-02	1.192
NDUFB3	NM_002491	4.81E-02	-1.226
CHMP5	NM_016410	5.19E-02	1.442
PSMD6	NM_001271781	5.25E-02	-1.509
TBCA	NM_004607	5.30E-02	1.192
RPL27	NM_000988	5.36E-02	-1.152
METTL5	NM_001293187	5.42E-02	1.436
RPS18	NM_022551	5.59E-02	1.141
ARL6IP5	NM_006407	5.79E-02	1.292
RIN2	NM_018993	5.84E-02	1.419
PPT1	NM_001142604	5.91E-02	1.447
NME1	NM_000269	5.99E-02	-1.406
EIF2S3	NM_001415	6.10E-02	1.292
VPS35	NM_018206	6.16E-02	1.308
PRDX3	NM_006793	6.48E-02	-1.303
ATP5H	NM_006356	6.79E-02	-1.289

PSMB5	NM_001130725	6.80E-02	-1.254
MYL12B	NM_033546	6.85E-02	-1.180
ARL1	NM_001177	6.95E-02	1.306
SPCS2	NM_014752	6.98E-02	1.279
PNRC2	NM_017761	7.21E-02	1.380
POMP	NM_015932	7.21E-02	1.226
SELK	NM_021237	7.23E-02	1.236
CD63	NM_001257400	7.41E-02	-1.276
GDI2	NM_001494	7.43E-02	1.434
SHFM1	NM_006304	7.56E-02	-1.212
PPA2	NM_176869	7.58E-02	-1.569
PRDX4	NM_006406	7.63E-02	-1.308
SDCBP	NM_005625	7.74E-02	1.362
DPYSL2	NM_001244604	7.81E-02	1.256
SCG5	NM_003020	7.87E-02	-1.144
SCG5	NM_001144757	8.16E-02	-1.139
ATP1B1	NM_001677	8.30E-02	-1.325
BCAS2	NM_005872	8.37E-02	1.463
DBI	NM_001079862	8.43E-02	1.210
FKBP2	NM_004470	8.50E-02	-1.391
C18orf32	NM_001199346	8.74E-02	1.291
SDCBP	NM_001007069	8.91E-02	1.421
EIF3H	NM_003756	9.05E-02	1.318
C3orf14	NM_001291941	9.12E-02	1.265
FKBP3	NM_002013	9.23E-02	-1.339
CNOT7	NM_001322091	9.59E-02	1.346
TUBA1B	NM_006082	9.69E-02	-1.161
NDUFC2	NM_004549	9.85E-02	-1.212
IRF2BP2	NM_182972	1.02E-01	1.310
RPS10	NM_001014	1.02E-01	1.175
NAP1L1	NM_001307924	1.05E-01	1.353
SLC39A9	NM_001252151	1.07E-01	1.389
RPL8	NM_000973	1.07E-01	-1.156
CHP1	NM_007236	1.11E-01	-1.203
RPL36	NM_033643	1.11E-01	1.127
COX7C	NM_001867	1.12E-01	-1.139
IRF2BP2	NM_001077397	1.12E-01	1.312
TMEM258	NM_014206	1.16E-01	-1.188
ARL8B	NM_018184	1.17E-01	1.393
SLC39A9	NM_001252152	1.17E-01	1.384
PSMA1	NM_002786	1.20E-01	1.242

PPIB	NM_000942	1.21E-01	-1.190
RPL9	NM_000661	1.23E-01	1.169
SKP1	NM_170679	1.25E-01	1.130
PRDX5	NM_012094	1.25E-01	-1.253
MORF4L1	NM_001265603	1.25E-01	1.143
VDAC3	NM_005662	1.26E-01	1.418
PPIG	NM_004792	1.26E-01	-1.330
ANXA7	NM_001156	1.27E-01	-1.255
SNRPE	NM_003094	1.28E-01	-1.277
MRPL51	NM_016497	1.29E-01	1.189
EIF4G2	NM_001418	1.32E-01	1.228
COPS8	NM_006710	1.34E-01	1.348
C11orf58	NM_014267	1.34E-01	1.169
SOD1	NM_000454	1.35E-01	-1.139
DYNLT1	NM_006519	1.35E-01	-1.212
MMADHC	NM_015702	1.37E-01	1.252
CALM1	NM_006888	1.39E-01	-1.136
ID2	NM_002166	1.40E-01	1.337
HSPA8	NM_006597	1.40E-01	1.271
HSPA5	NM_005347	1.41E-01	1.742
MRPL39	NM_017446	1.43E-01	-1.421
ATP5G2	NM_005176	1.47E-01	-1.227
SARS	NM_006513	1.48E-01	1.292
TAX1BP1	NM_001079864	1.49E-01	1.221
GPX4	NM_002085	1.54E-01	-1.236
COPZ1	NM_016057	1.58E-01	-1.199
RPL19	NM_000981	1.59E-01	1.115
GTF2A2	NM_004492	1.59E-01	1.274
TMED2	NM_006815	1.61E-01	1.232
SCGN	NM_006998	1.63E-01	1.213
CHCHD2	NM_016139	1.66E-01	-1.204
SEC61B	NM_006808	1.68E-01	-1.138
COX7A2L	NM_004718	1.70E-01	1.204
NDUFAB1	NM_005003	1.73E-01	-1.239
SBDS	NM_016038	1.73E-01	1.220
NDUFB9	NM_005005	1.75E-01	-1.185
REEP5	NM_005669	1.76E-01	1.231
ARPC5	NM_005717	1.77E-01	-1.208
TSPYL5	NM_033512	1.78E-01	1.344
NDUFB6	NM_002493	1.80E-01	-1.231
ATPIF1	NM_016311	1.82E-01	-1.193

TSPYL1	NM_003309	1.86E-01	1.145
EIF3I	NM_003757	1.86E-01	-1.241
HACD3	NM_016395	1.87E-01	1.270
LINC01578	NR_037602	1.89E-01	1.261
LAMTOR4	NM_001008395	1.91E-01	-1.243
CALM2	NM_001305625	1.93E-01	1.158
RPLP0	NM_001002	1.95E-01	1.106
RPS19	NM_001022	1.96E-01	-1.150
EIF4E	NM_001130678	2.00E-01	1.178
SELT	NM_016275	2.00E-01	1.197
NDUFA12	NM_018838	2.08E-01	1.171
DNAJA1	NM_001539	2.08E-01	-1.190
MDH1	NM_001316374	2.09E-01	-1.182
MDH1	NM_005917	2.09E-01	-1.182
H3F3A	NM_002107	2.09E-01	-1.163
RPS19	NM_001321483	2.10E-01	1.143
YWHAQ	NM_006826	2.10E-01	1.172
ATP6V1D	NM_015994	2.14E-01	1.178
UBC	NM_021009	2.16E-01	1.140
DSTN	NM_006870	2.16E-01	1.128
SYPL1	NM_182715	2.25E-01	1.298
RPL18	NM_000979	2.25E-01	1.143
FOS	NM_005252	2.25E-01	-1.257
C14orf166	NM_016039	2.26E-01	1.210
PSMA3	NM_002788	2.33E-01	1.201
UBL5	NM_001048241	2.34E-01	-1.124
NDUFS5	NM_001184979	2.40E-01	-1.144
NUCB2	NM_005013	2.42E-01	1.165
HSP90AB1	NM_007355	2.46E-01	1.100
ATP6V0B	NM_004047	2.48E-01	-1.181
RPS8	NM_001012	2.51E-01	1.120
EEF1E1	NM_004280	2.53E-01	1.303
SARAF	NM_016127	2.54E-01	1.225
ATP5F1	NM_001688	2.55E-01	-1.166
COPB2	NM_004766	2.59E-01	1.227
UBB	NM_001281720	2.59E-01	-1.215
BTF3	NM_001207	2.61E-01	1.158
EEF1A1	NM_001402	2.63E-01	1.065
SSR1	NM_003144	2.65E-01	1.165
RUFY3	NM_001291993	2.65E-01	-1.203
CAPZA2	NM_006136	2.66E-01	-1.152

ATP6V1F	NM_004231	2.68E-01	-1.169
HSPD1	NM_199440	2.72E-01	1.175
TUBA4A	NM_006000	2.74E-01	-1.215
PFN2	NM_053024	2.75E-01	-1.278
ACTB	NM_001101	2.76E-01	-1.246
42628	NM_004261	2.77E-01	1.191
ETNK1	NM_018638	2.82E-01	-1.135
SEC61G	NM_014302	2.82E-01	1.138
WDR83OS	NM_016145	2.83E-01	-1.177
ARPC3	NM_001278556	2.86E-01	-1.121
PSMB4	NM_002796	2.87E-01	-1.150
CPNE3	NM_003909	2.90E-01	-1.175
EMC4	NM_016454	2.90E-01	-1.225
ATP6V1B2	NM_001693	2.92E-01	1.265
HSPD1	NM_002156	2.98E-01	1.159
BZW1	NM_001321688	2.99E-01	-1.188
MRPL13	NM_014078	3.02E-01	-1.209
RPS11	NM_001015	3.06E-01	1.100
TPI1	NM_000365	3.13E-01	-1.112
ANP32E	NM_001136479	3.20E-01	1.165
TIMP1	NM_003254	3.23E-01	-1.317
N4BP2L2	NM_014887	3.28E-01	-1.187
RPS27A	NM_002954	3.30E-01	1.102
GABARAPL2	NM_007285	3.30E-01	1.143
MRPL42	NM_014050	3.33E-01	1.175
ARRDC3	NM_001329672	3.42E-01	1.146
MRPL50	NM_019051	3.50E-01	1.192
TAF9B	NM_015975	3.58E-01	1.246
PARM1	NM_015393	3.64E-01	1.111
SNRPD2	NM_004597	3.66E-01	-1.101
PTPRN	NM_001199764	3.71E-01	-1.163
EFNA5	NM_001962	3.71E-01	-1.172
PSMC6	NM_002806	3.71E-01	1.215
B2M	NM_004048	3.75E-01	-1.171
SSBP1	NM_003143	3.76E-01	-1.143
VPS29	NM_057180	3.78E-01	-1.177
SUB1	NM_006713	3.79E-01	1.109
LRRC75A-AS1	NR_027166	3.92E-01	1.127
PPA1	NM_021129	3.93E-01	-1.138
COX4I1	NM_001861	3.98E-01	-1.091
RPL35	NM_007209	3.98E-01	-1.098

UQCR10	NM_013387	4.01E-01	-1.113
PCBP1	NM_006196	4.01E-01	1.159
SPCS1	NM_014041	4.04E-01	1.091
JKAMP	NM_016475	4.06E-01	1.202
CFAP36	NM_080667	4.09E-01	1.170
C6orf62	NM_030939	4.09E-01	1.089
TAPBP	NM_172209	4.10E-01	-1.150
ATP5L	NM_006476	4.13E-01	-1.131
COPB1	NM_001144062	4.17E-01	-1.167
RPL36AL	NM_001001	4.19E-01	-1.073
C1GALT1C1	NM_001011551	4.19E-01	-1.196
ESD	NM_001984	4.21E-01	-1.138
VDAC2	NM_001184823	4.21E-01	1.140
PSMA4	NM_002789	4.29E-01	-1.148
LMBRD1	NM_018368	4.35E-01	1.145
COX5B	NM_001862	4.35E-01	-1.119
EDF1	NM_003792	4.36E-01	-1.114
NOP58	NM_015934	4.40E-01	1.113
SLC30A8	NM_001172814	4.40E-01	1.156
SEC22B	NM_004892	4.41E-01	1.077
ATP5C1	NM_001001973	4.43E-01	-1.108
PEMT	NM_148173	4.44E-01	-1.117
SRP14	NM_003134	4.44E-01	1.067
SAR1A	NM_020150	4.48E-01	-1.079
MYL12A	NM_001303048	4.49E-01	1.164
RAB1A	NM_004161	4.52E-01	-1.143
SSB	NM_003142	4.61E-01	1.130
COX6C	NM_004374	4.67E-01	-1.062
PPT1	NM_000310	4.70E-01	1.159
SF3B5	NM_031287	4.70E-01	-1.122
ATP5J	NM_001003697	4.71E-01	-1.076
DDX5	NM_001320597	4.71E-01	-1.084
ARF1	NM_001658	4.77E-01	-1.115
PSMD7	NM_002811	4.78E-01	1.114
PAPOLA	NM_001252006	4.85E-01	1.167
SSR2	NM_003145	4.86E-01	-1.112
CSDE1	NM_007158	4.91E-01	1.127
SAR1A	NM_001142648	5.02E-01	-1.083
PSMA2	NM_002787	5.07E-01	1.097
EGR1	NM_001964	5.10E-01	-1.136
RPS7	NM_001011	5.11E-01	-1.072

SLIRP	NM_031210	5.13E-01	-1.067
TXN	NM_003329	5.16E-01	-1.083
NDUFA6	NM_002490	5.19E-01	-1.093
COX14	NM_032901	5.28E-01	1.091
RPL38	NM_000999	5.32E-01	-1.063
RPL18A	NM_000980	5.37E-01	1.051
GABARAP	NM_007278	5.39E-01	1.054
RPS24	NM_001142282	5.45E-01	-1.067
RPL23A	NM_000984	5.51E-01	1.043
SRSF3	NM_003017	5.55E-01	-1.065
TUBA1A	NM_006009	5.58E-01	1.095
TMSB4X	NM_021109	5.65E-01	1.082
BNIP3	NM_004052	5.68E-01	-1.125
42620	NM_001011553	5.68E-01	1.073
TXLNA	NM_175852	5.69E-01	1.104
ARF6	NM_001663	5.74E-01	-1.115
IER3IP1	NM_016097	5.87E-01	-1.087
SF3B6	NM_016047	5.92E-01	-1.077
PRELID3B	NM_001256403	5.93E-01	-1.081
PABPC1	NM_002568	5.94E-01	1.086
RPLP1	NM_001003	5.98E-01	1.037
RPL10A	NM_007104	5.99E-01	1.073
SUMO1	NM_003352	6.03E-01	1.086
UQCRH	NM_006004	6.08E-01	-1.082
CAPZA1	NM_006135	6.18E-01	-1.057
MRPS10	NM_018141	6.19E-01	1.093
PIGP	NM_153682	6.22E-01	-1.103
ACTR10	NM_018477	6.22E-01	1.102
TM9SF2	NM_004800	6.24E-01	1.086
SF3B1	NM_012433	6.25E-01	-1.090
HAT1	NM_003642	6.26E-01	1.147
TMEM208	NM_014187	6.26E-01	1.092
ANAPC13	NM_015391	6.28E-01	-1.087
XRCC5	NM_021141	6.39E-01	1.050
SNHG6	NR_002599	6.42E-01	1.080
ACTG1	NR_037688	6.43E-01	1.099
UBE2N	NM_003348	6.44E-01	-1.070
TMEM230	NM_001009925	6.46E-01	1.088
COPS4	NM_016129	6.62E-01	-1.107
DNTTIP2	NM_014597	6.68E-01	-1.078
EIF3M	NM_006360	6.71E-01	-1.079

TMEM35B	NM_001195156	6.79E-01	1.070
BEX4	NM_001080425	6.82E-01	-1.076
YWHAZ	NM_001135700	6.89E-01	-1.053
SET	NM_001248001	6.90E-01	1.058
DNAJC12	NM_021800	6.98E-01	1.071
RTN4	NM_007008	7.02E-01	-1.078
MAPK1IP1L	NM_144578	7.03E-01	-1.050
RPS16	NM_001020	7.08E-01	-1.043
PEBP1	NM_002567	7.09E-01	1.051
ATRAID	NM_001170795	7.09E-01	-1.060
YPEL5	NM_016061	7.09E-01	1.081
PSMC5	NM_002805	7.18E-01	1.068
SNRPD1	NM_006938	7.21E-01	-1.055
DYNLL1	NM_003746	7.23E-01	-1.042
ANXA2	NM_004039	7.24E-01	1.157
MRPS33	NM_053035	7.24E-01	-1.067
CMC1	NM_182523	7.25E-01	-1.076
BEX5	NM_001012978	7.27E-01	1.087
CACYBP	NM_014412	7.30E-01	-1.068
SNRPN	NM_003097	7.31E-01	1.040
SNURF	NM_005678	7.31E-01	1.040
CACUL1	NM_153810	7.41E-01	1.051
DLD	NM_000108	7.45E-01	1.064
EPCAM	NM_002354	7.50E-01	1.050
DDX5	NM_001320596	7.56E-01	-1.034
UBE2D3	NM_181893	7.57E-01	1.054
VMA21	NM_001017980	7.65E-01	1.057
TMEM126B	NM_001256547	7.66E-01	-1.063
HSBP1	NM_001537	7.67E-01	1.035
RPL15	NM_002948	7.73E-01	1.031
NDUFB4	NM_004547	7.74E-01	1.030
PSMA6	NM_002791	7.78E-01	-1.041
MYL6	NM_021019	7.79E-01	-1.025
DDX5	NM_004396	7.80E-01	-1.040
RPS10-NUDT3	NM_001202470	7.80E-01	-1.054
RPL37A	NM_000998	7.81E-01	-1.022
NDUFB5	NM_002492	7.88E-01	1.058
PFN2	NM_002628	7.93E-01	-1.054
STRAP	NM_007178	7.93E-01	1.039
CLDND1	NM_001040181	7.93E-01	-1.049
FTL	NM_000146	7.97E-01	-1.067

RPS29	NM_001032	7.97E-01	-1.027
MRPS18C	NM_016067	7.98E-01	1.052
UBE2V2	NM_003350	8.02E-01	1.041
CCT8	NM_006585	8.03E-01	1.045
RPSA	NM_002295	8.03E-01	1.030
RPL39	NM_001000	8.15E-01	1.022
ZBED5-AS1	NR_034137	8.16E-01	-1.050
MRPL33	NM_145330	8.18E-01	-1.035
THUMPD1	NM_001304550	8.22E-01	1.039
ATP6V0E1	NM_003945	8.24E-01	-1.023
SRP9	NM_003133	8.33E-01	1.028
YWHAH	NM_003405	8.36E-01	-1.034
MPV17	NM_002437	8.41E-01	1.039
DPY30	NM_001321209	8.42E-01	-1.033
GAPDH	NM_001289746	8.51E-01	1.029
DAZAP2	NM_014764	8.54E-01	1.037
ITM2B	NM_021999	8.64E-01	1.020
THOC2	NM_001081550	8.70E-01	1.025
TMED10	NM_006827	8.70E-01	1.020
MAPRE1	NM_012325	8.70E-01	-1.025
PSMD14	NM_005805	8.73E-01	-1.029
LSM3	NM_014463	8.78E-01	1.021
ATP5O	NM_001697	8.82E-01	-1.019
RPL29	NM_000992	8.87E-01	1.017
STMN2	NM_007029	8.89E-01	1.031
HNRNPA1	NM_002136	8.93E-01	1.017
CNIH1	NM_005776	8.98E-01	1.024
TSPAN7	NM_004615	9.12E-01	-1.020
RPLP2	NM_001004	9.22E-01	1.010
IMMP1L	NM_001304274	9.25E-01	-1.027
MDH1	NM_001199111	9.27E-01	-1.012
TOMM5	NM_001001790	9.31E-01	-1.014
PTGES3	NM_006601	9.31E-01	-1.015
HNRNPH1	NM_005520	9.33E-01	-1.009
CMC2	NM_020188	9.36E-01	-1.016
YWHAB	NM_139323	9.36E-01	-1.011
MIF	NM_002415	9.38E-01	-1.010
SMC3	NM_005445	9.43E-01	-1.012
AK6	NM_016283	9.43E-01	1.013
RPL7A	NM_000972	9.47E-01	1.006
COX7B	NM_001866	9.51E-01	1.007

YWHAZ	NM_145690	9.52E-01	-1.009
SUMO2	NM_006937	9.65E-01	1.005
HMGB1	NM_002128	9.66E-01	-1.005
ZC3H15	NM_018471	9.71E-01	1.007
PDCD5	NM_004708	9.77E-01	1.005
TUBB	NM_178014	9.79E-01	1.004
LAMTOR5	NM_006402	9.80E-01	1.003
PAFAH1B2	NR_110282	9.81E-01	1.003
HNRNPH1	NM_001257293	9.93E-01	-1.001
PIGF	NM_002643	9.98E-01	1.001
EID1	NM_014335	1.00E+00	-1.000

Supplementary Figures

Supplementary figure 1: Diagrams of C1 wells in the study with A) 1 single cell captured, B) no cell captured and C) more than 1 cell captured. All wells without any cells or with multiple cells captured were excluded from further analysis.

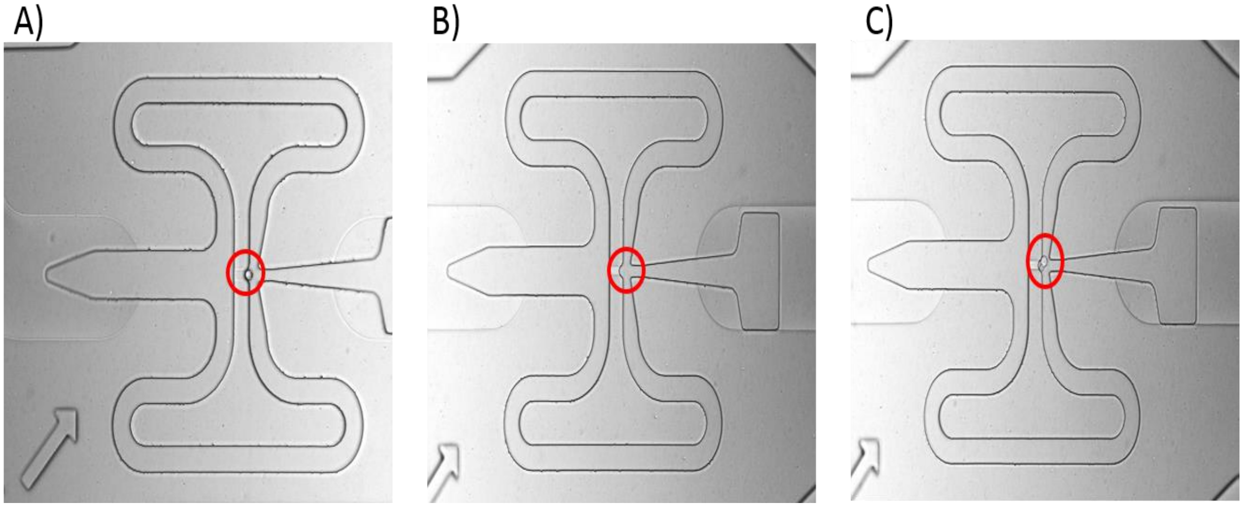

Supplementary Figure 2: Mapping of sequence read to exonic, intergenic and intronic regions among 442 single cells captured in the study. 47 cells with < 30% mapping to exonic regions were excluded.

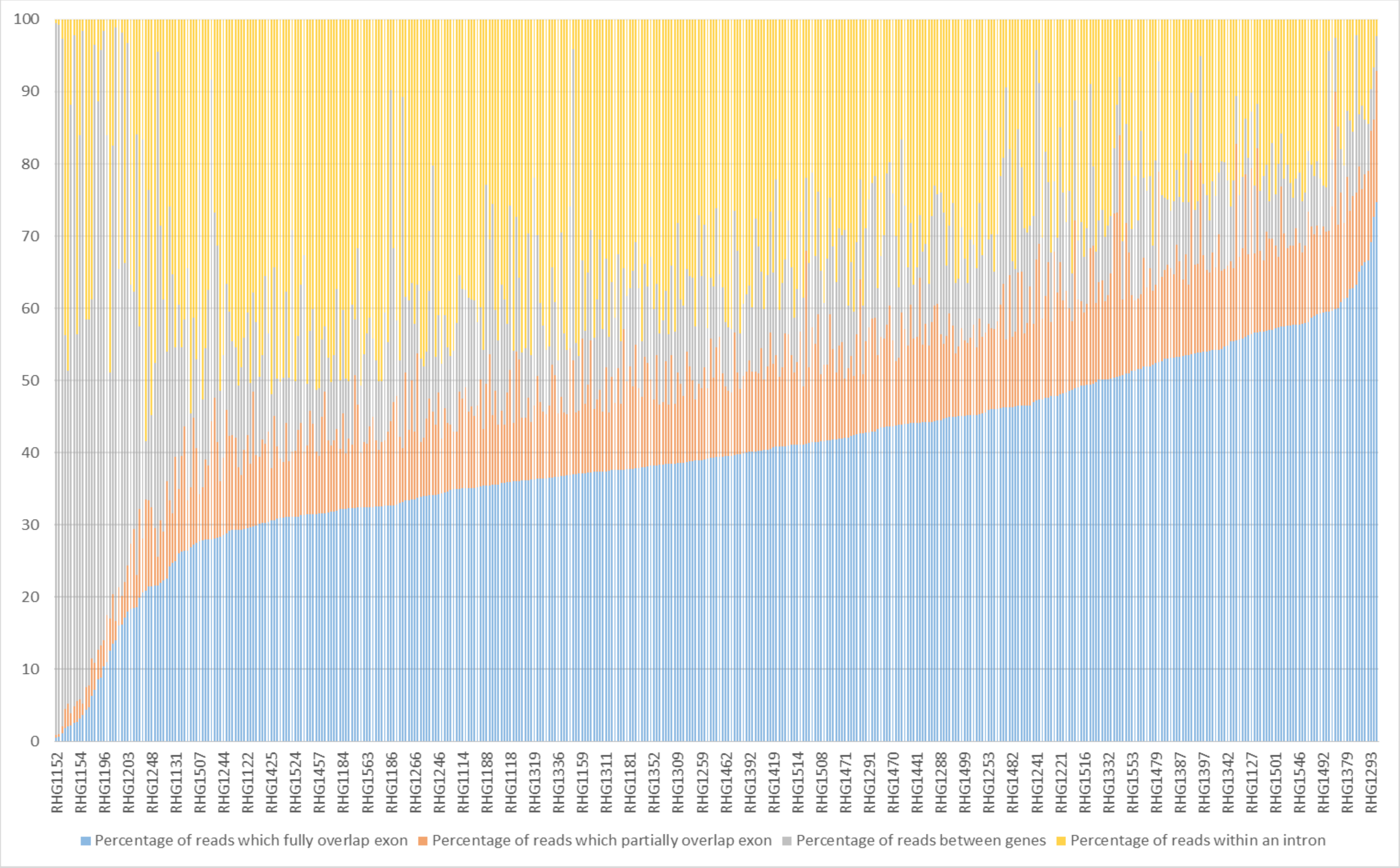

Supplementary Figure 3: Clustering of 294 single islet cells based on all 252 transcripts (146 genes) with RPKM values ≥ 500 in ≥ 2% of cells revealed 3 main cluster of cells. These were based on *GCG* (green oval), *INS* (red oval) and *REG1A* (yellow oval). Scale indicates quantile-normalized expression levels.

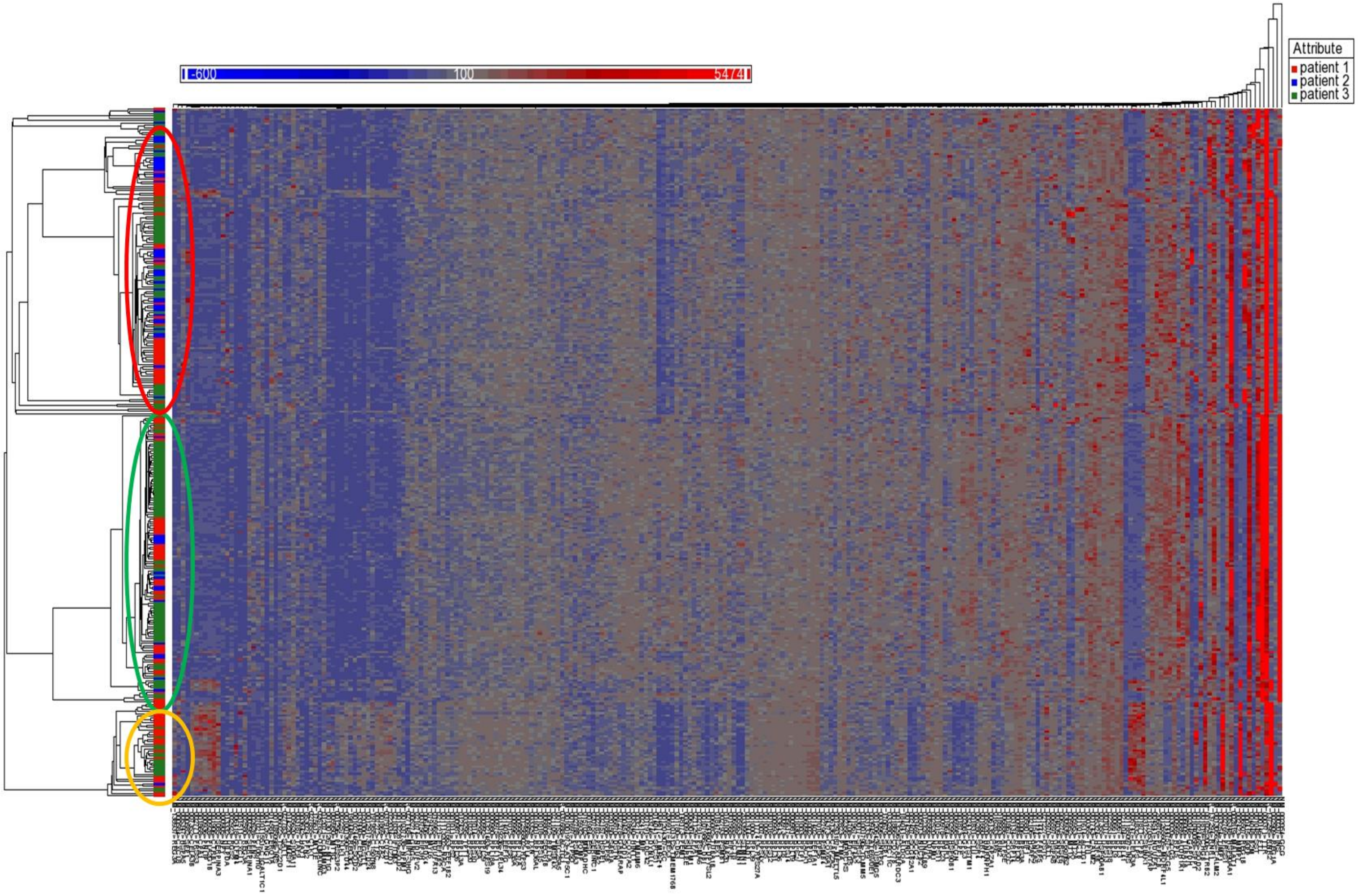

Supplementary Figure 4: Hierarchical clustering of 294 single islet cells based on pancreatic cell lineage gene transcripts and *REG1A*. 10 transcripts (from 10 genes) with RPKM values > 500 in 25% of cells were utilized for clustering and data was quantile normalized. 18 doublet cells indicated in yellow boxes. Red oval: cluster in INS expressing cells; blue oval: cluster of SST expressing cells; green oval: cluster of GCG expressing cells; orange oval: cluster of REG1A expressing cells. Scale indicates quantile-normalized expression levels.

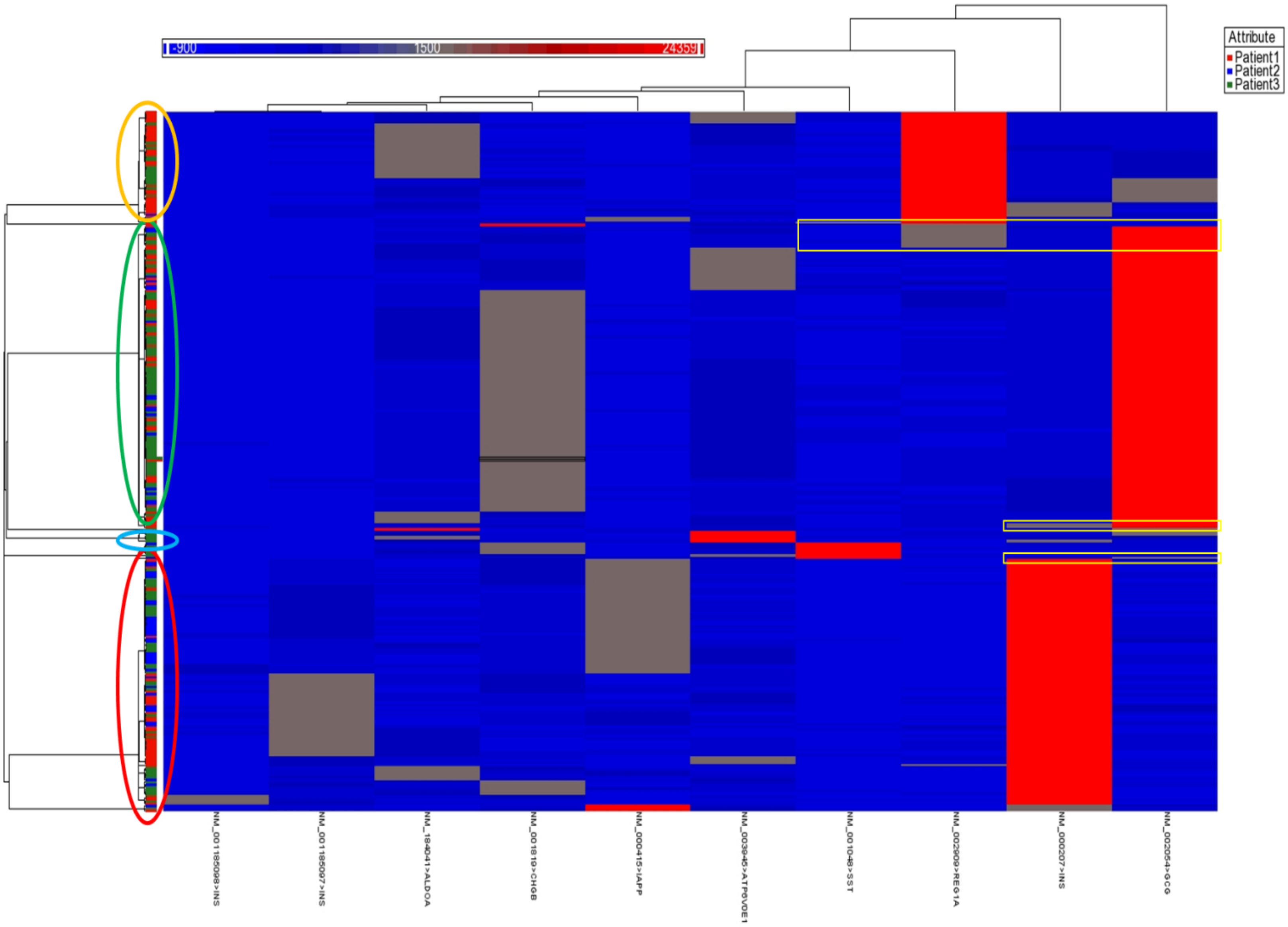

Supplementary Figure 5: Hierarchical clustering 226 predominantly mono-hormonal cells among our based on *INS*, *GCG* and *SST* expression levels. Scale indicates quantile-normalized expression levels.

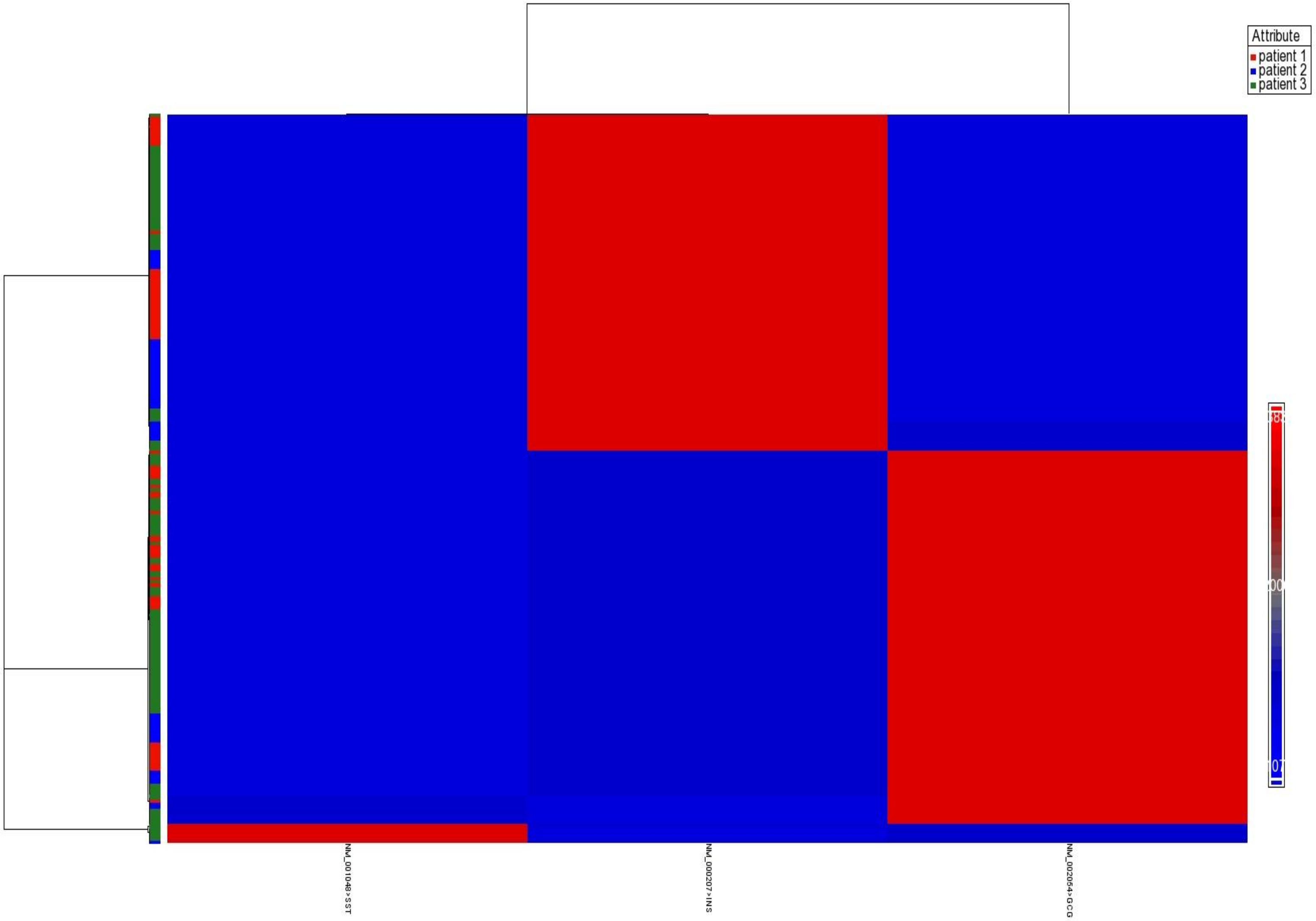

Supplementary figure 6: Hierarchical clustering of 118 *GCG* expressing cells using transcripts with RPKM values ≥ 500 in ≥ 2% of cells. We however did not identify distinct sub-clustering of these *GCG* and *INS* cells, although some cells were separated based on relative expression on *TTR*. Repeating these analyses using less stringent transcript RPKM cut-off (RPKM values ≥ 50 in ≥ 2% of cells) did not change main findings (data not shown)

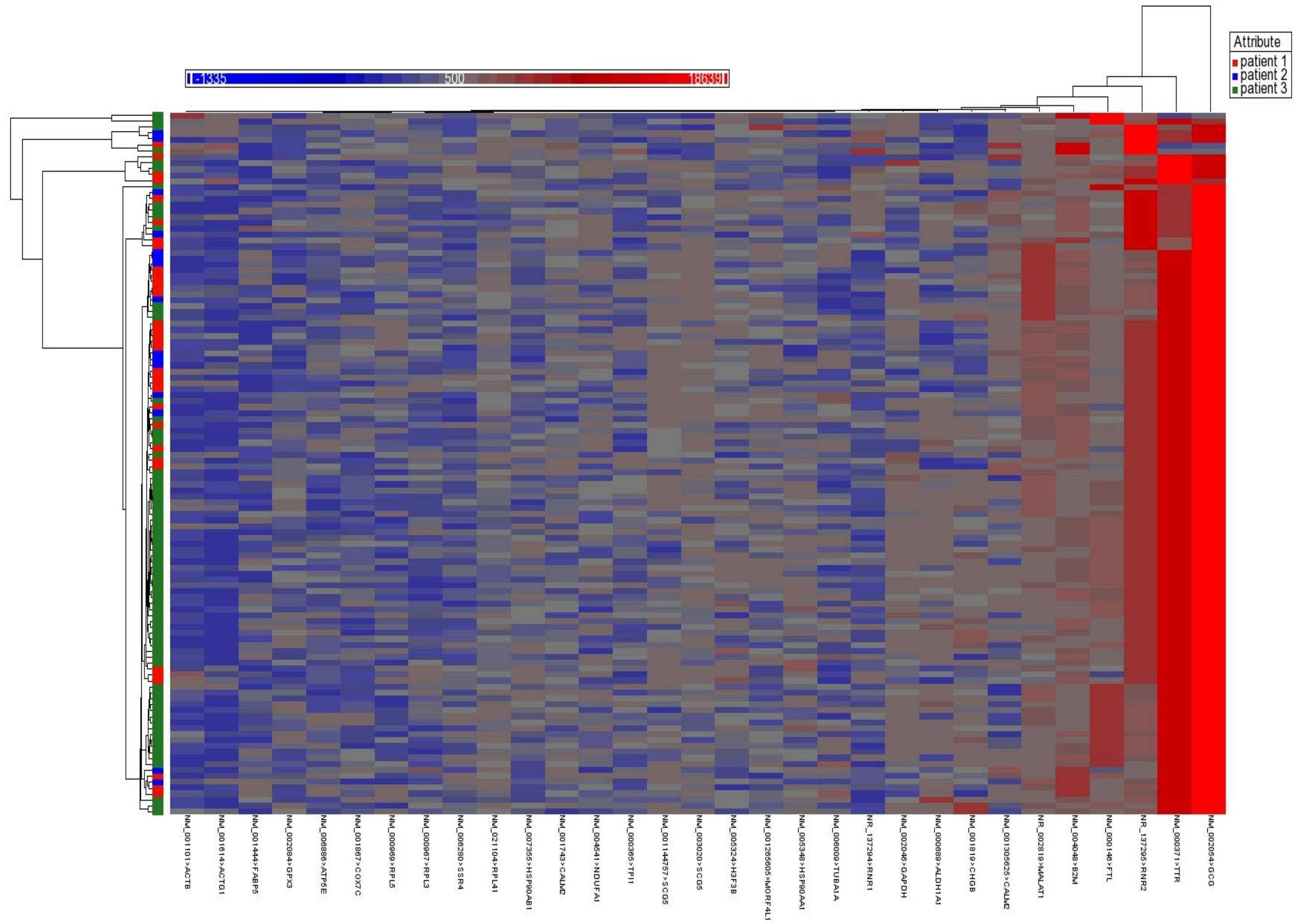

Supplementary figure 7: Hierarchical clustering of 105 *INS* expressing cells using transcripts with RPKM values ≥ 500 in ≥ 2% of cells. We however did not identify distinct sub-clustering of these *GCG* and *INS* cells, although some cells were separated based on relative expression on *RNR2*. Repeating these analyses using less stringent transcript RPKM cut-off (RPKM values ≥ 50 in ≥ 2% of cells) did not change main findings (data not shown)

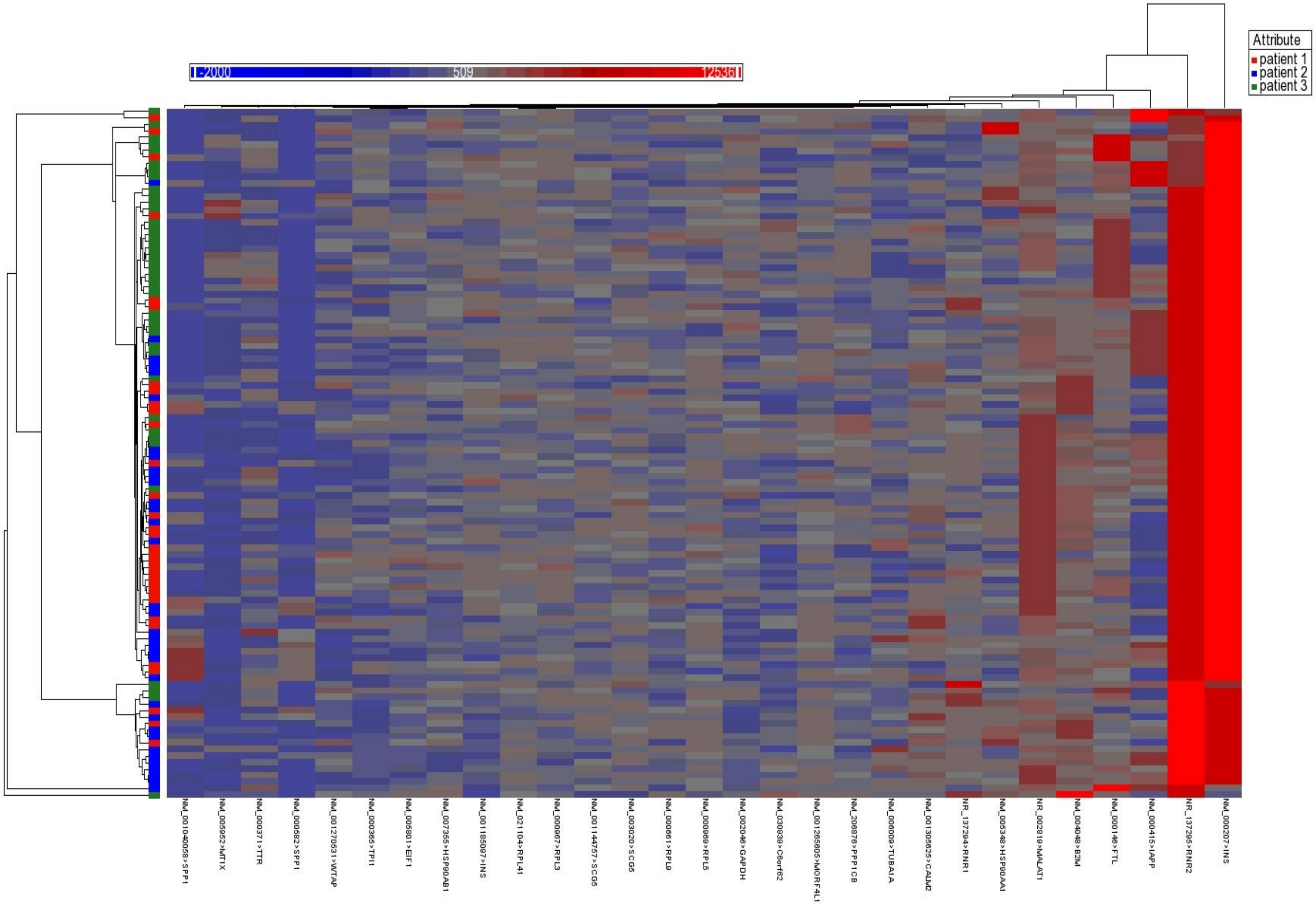

Supplementary figure 8: Number of transcripts and genes detected at each of the 276 cells that passed QC.

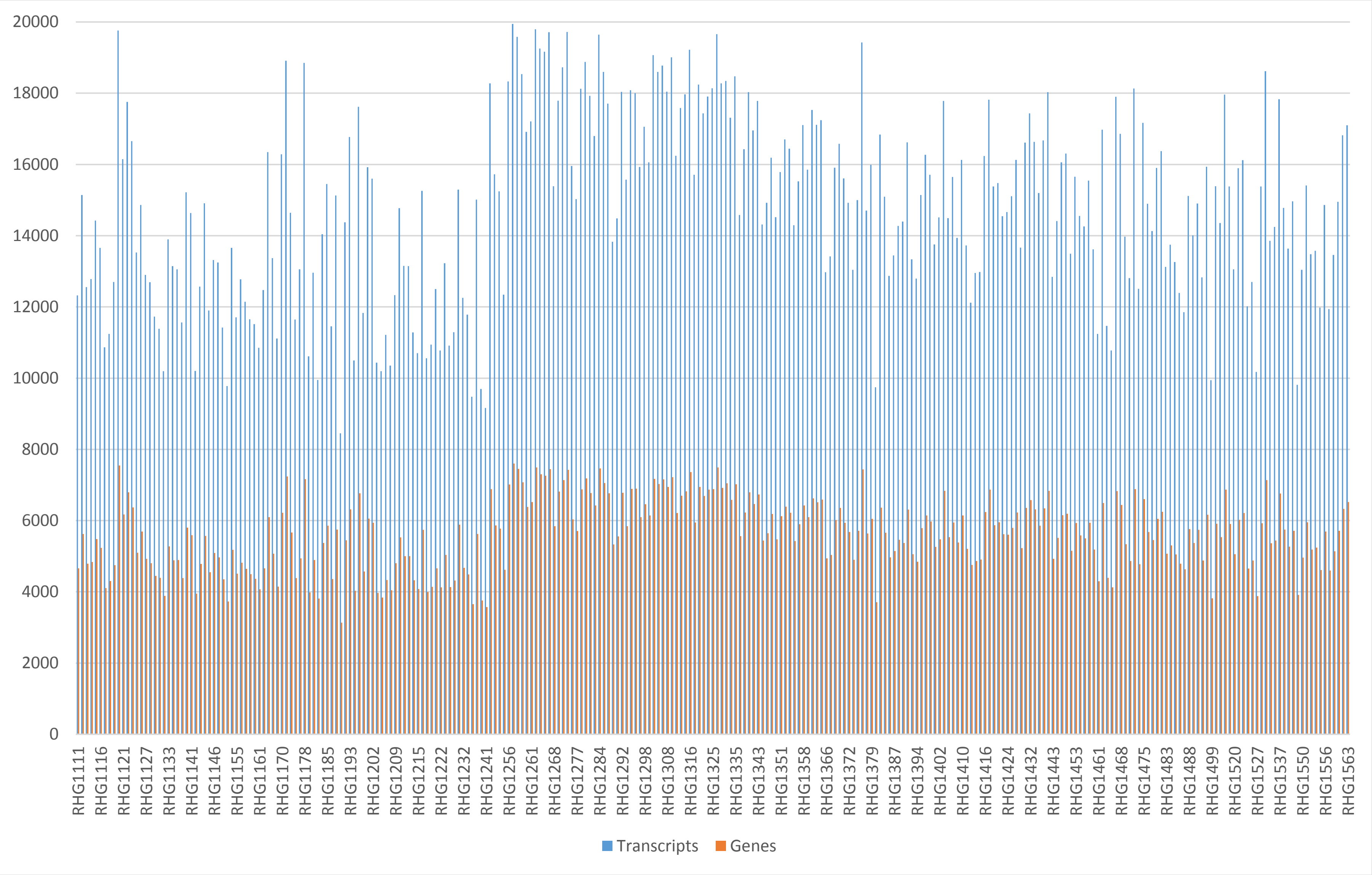

Supplementary figure 9: A) 5 significant canonical pathways identified from 11 significant β-cell genes. B) 2 significant canonical pathways identified from 17 significant α-cell genes. Threshold line represent p-value cut-off for significant pathways after Benjamini-Hochberg correction. Ratio represent proportion of input genes in canonical pathway.

A)

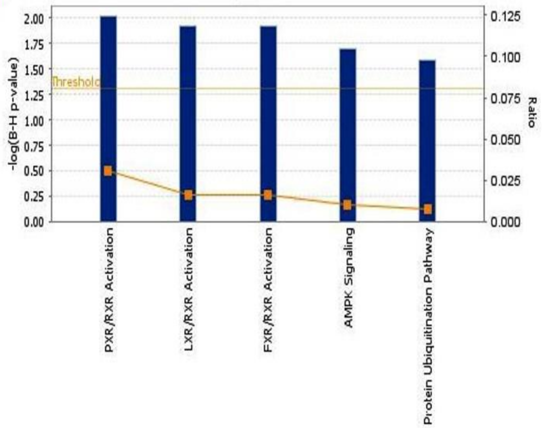

Ingenuity Canonical Pathways	-log(B-H p-value)	Molecules
PXR/RXR Activation	2.02	SCD,INS
LXR/RXR Activation	1.92	SCD,HADH
FXR/RXR Activation	1.92	G6PC2,INS
AMPK Signaling	1.70	INS,PFKFB2
Protein Ubiquitination Pathway	1.59	UCHL1,DNAJC3

B)

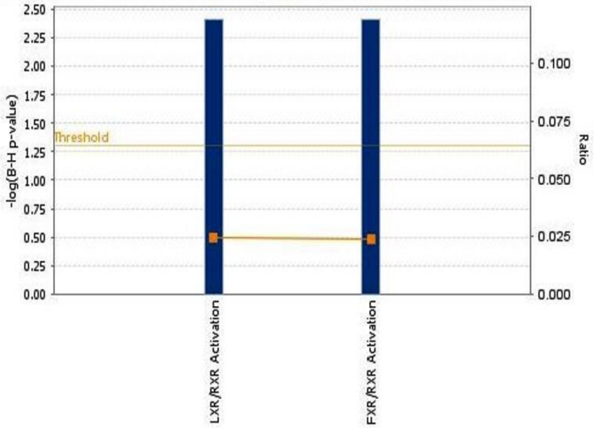

Ingenuity Canonical Pathways	-log(B-H p-value)	Molecules
LXR/RXR Activation	2.41	TTR,GC,CLU
FXR/RXR Activation	2.41	TTR,GC,CLU

Supplementary 10: Additional immunofluorescence sections showing co-localization of DNAJC3 with INS+ cells from pancreas of three different subjects. Subject 1 (1A-D), subject 2 (1E-H) and subject 3 (1I-L). DNAJC3 (green), INS (red), GCG (blue) and nucleus (grey) stains. Cell co-expressing INS and DNAJC indicated in yellow. Scale bar represents 20µm.

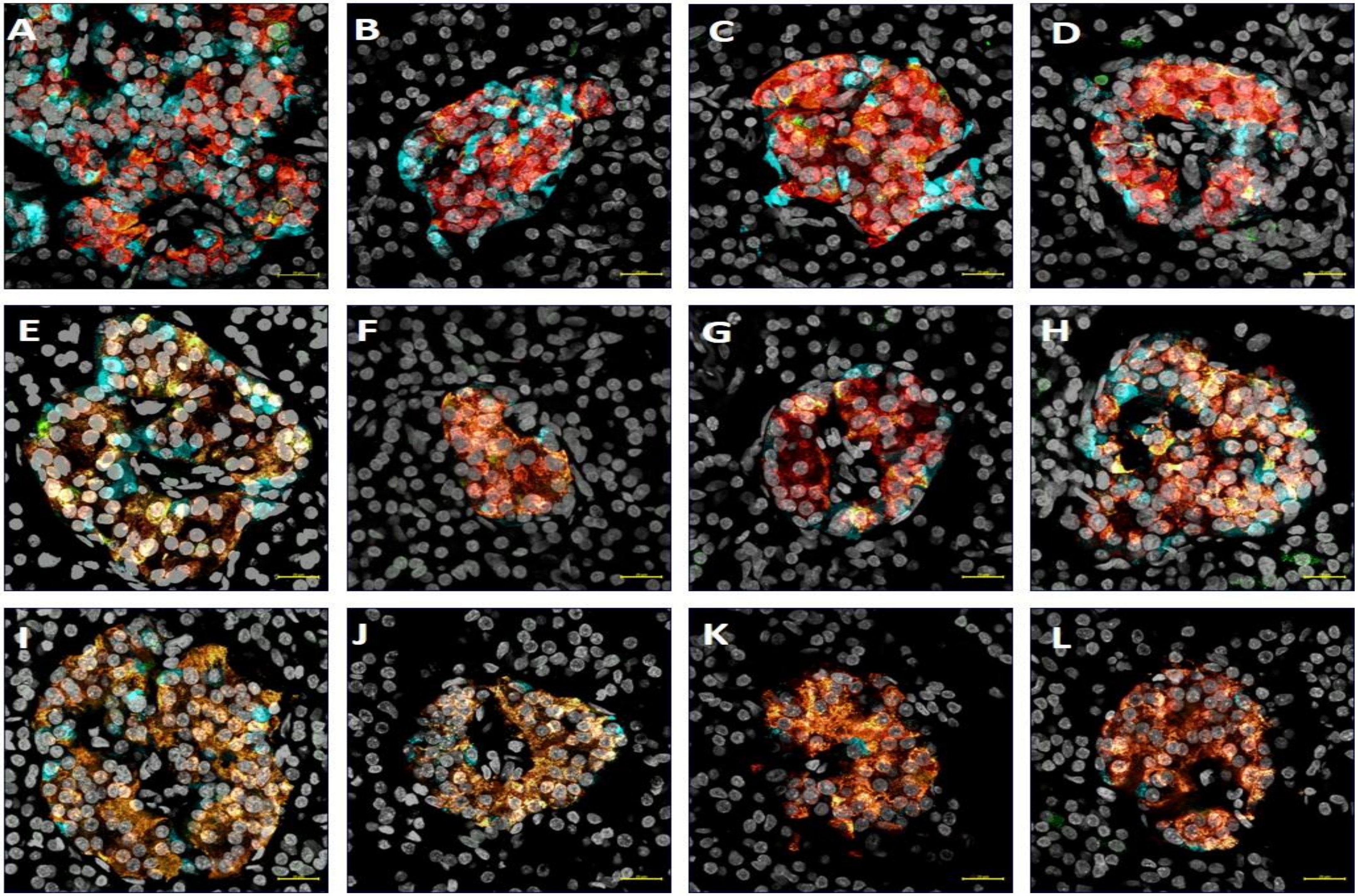

Supplementary 11: Additional immunofluorescence sections showing co-localization of PFKB2 with INS+ cells from pancreas of three different subjects. Subject 1 (1A-D), subject 2 (1E-H) and subject 3 (1I-L). PFKB2 (green), INS (red), GCG (blue) and nucleus (grey) stains. Cell co-expressing INS and PFKB2 indicated in yellow. Scale bar represents 20µm.

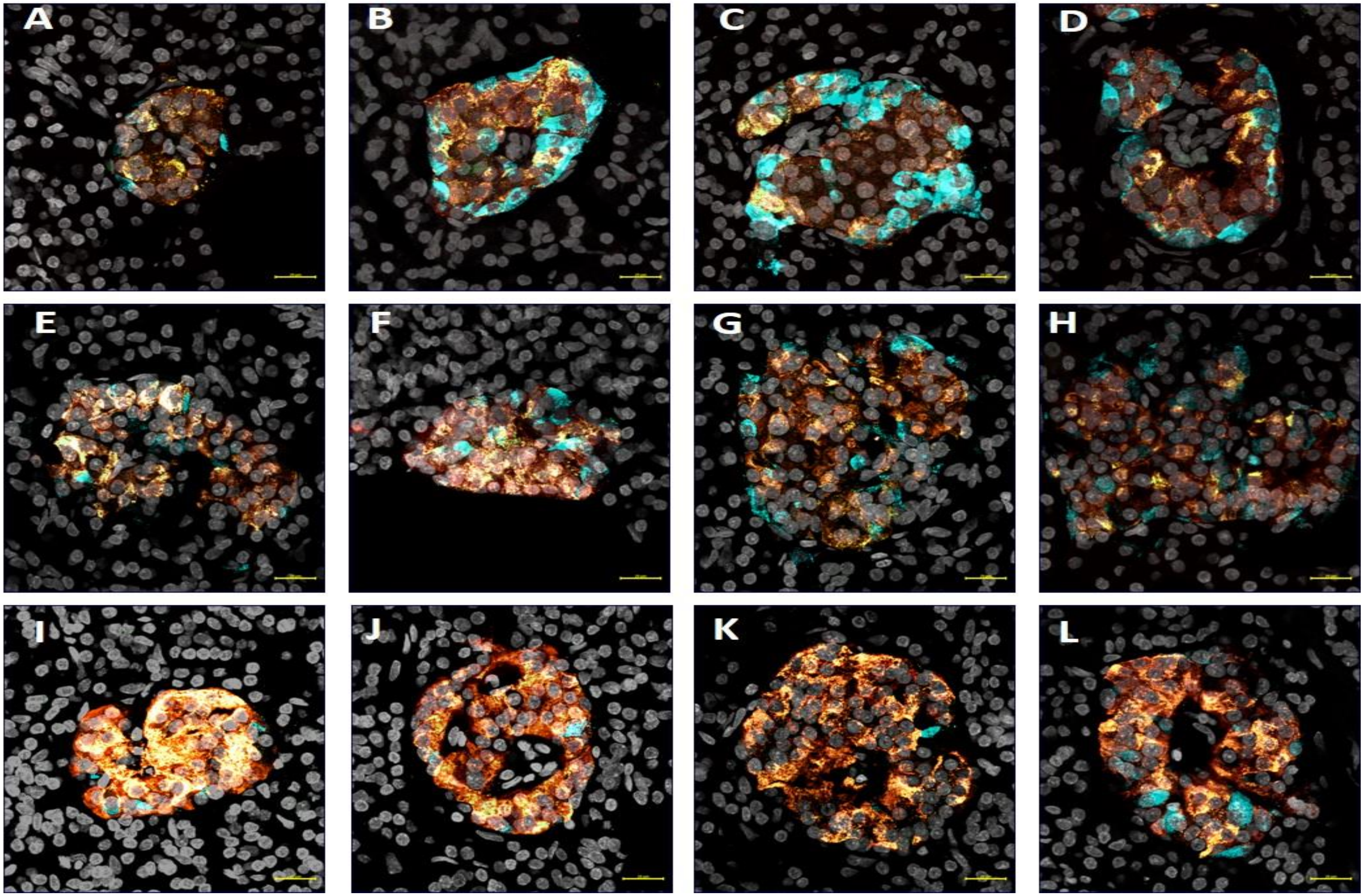

Supplementary 12: Additional immunofluorescence sections showing co-localization of UCHL1 with INS+ cells from pancreas of three different subjects. Subject 1 (1A-D), subject 2 (1E-H) and subject 3 (1I-L). UCHL1 (green), INS (red) and nucleus (grey) stains. Cell co-expressing INS and UCHL1 indicated in yellow. Scale bar represents 20µm.

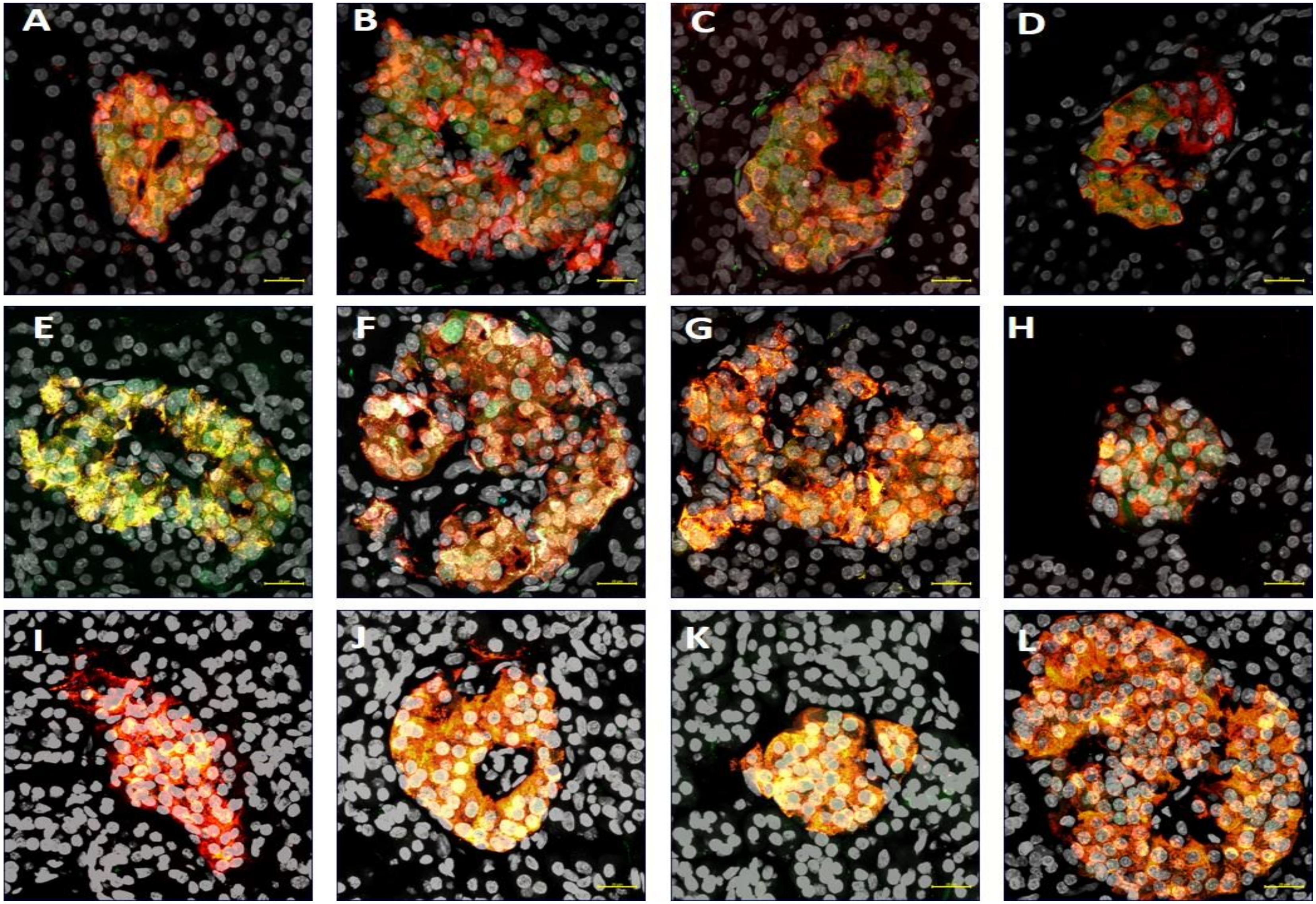

Supplementary figure 13: A) 3 significant canonical pathways identified from 16 common α-cell genes. B) 6 significant canonical pathways identified from 14 common β-cell genes. Threshold line represent p-value cut-off for significant pathways after Benjamini-Hochberg correction. Ratio represent proportion of input genes in canonical pathway.

A)

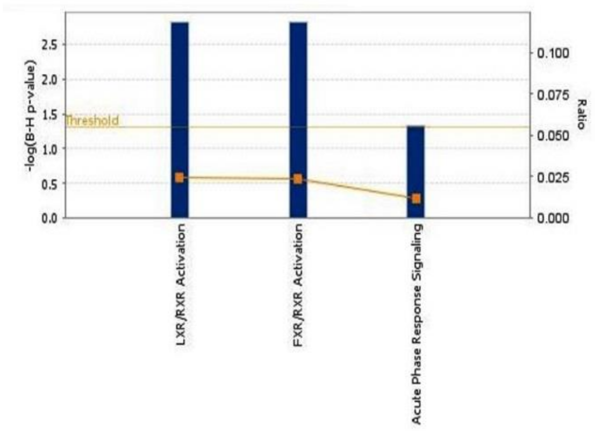

Ingenuity Canonical Pathways	-log(B-H p-value)	Molecules
LXR/RXR Activation	2.83	TTR,SERPINA1,GC
FXR/RXR Activation	2.83	TTR,SERPINA1,GC
Acute Phase Response Signaling	1.32	TTR,SERPINA1

B)

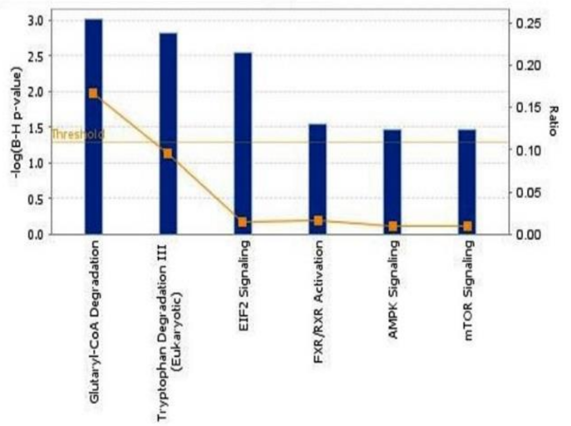

Ingenuity Canonical Pathways	-log(B-H p-value)	Molecules
Glutaryl-CoA Degradation	3.01	ACAT1,HADH
Tryptophan Degradation III (Eukaryotic)	2.81	ACAT1,HADH
EIF2 Signaling	2.54	RPL3,INS,EIF4A2
FXR/RXR Activation	1.56	G6PC2,INS
AMPK Signaling	1.47	INS,PFKFB2
mTOR Signaling	1.47	INS,EIF4A2

Supplementary figure 14: Canonical pathways detected from Ingenuity pathway analysis of unique β-cell genes (significantly high in only our study). No pathways remained significant after p-value corrections from unique α-cell gene list. Threshold line represent p-value cut-off for significant pathways after Benjamini-Hochberg correction. Ratio represent proportion of input genes in canonical pathway.

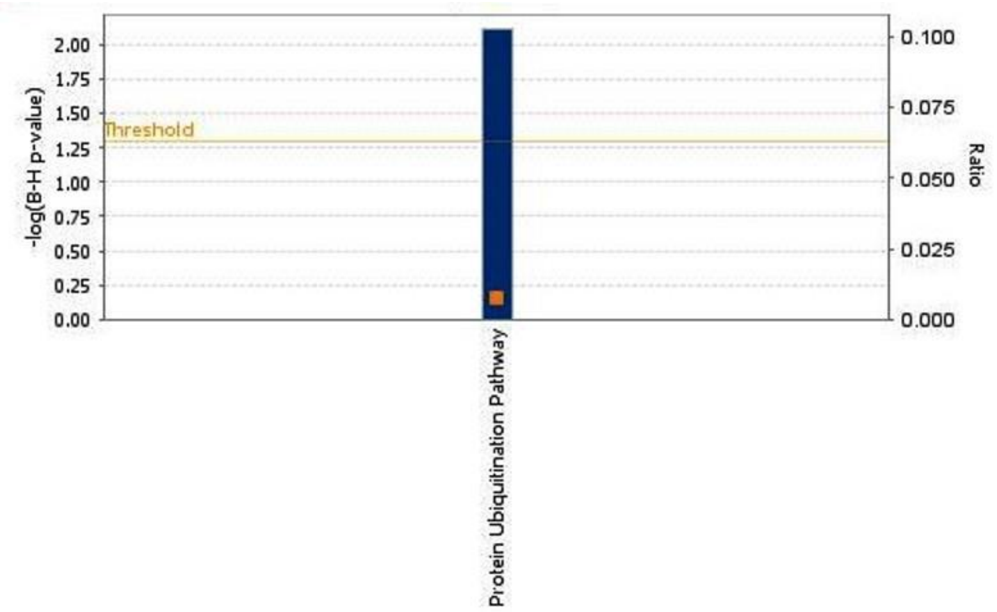

Ingenuity Canonical Pathways	-log(B-H p-value)	Molecules
Protein Ubiquitination Pathway	2.11	UCHL1,DNAJC3

Supplementary figure 15: Protein ubiquitination pathway identified to be significantly associated in East-Asian β-cells. Gene highlighted in green: UCHL1. Gene highlighted in orange: DNAJC3. Canonical pathway analysis performed using Ingenuity Pathway Analysis (version 1-07). Mean (SD) RPKM of UCHL1 in β-cells = 66.8 (66.9), Mean (SD) RPKM of UCHL1 in α-cells = 25.6 (41.6), Mean (SD) RPKM of DNAJC3 in β-cells = 134.3 (136.7) and Mean (SD) RPKM of DNAJC3 in α-cells = 70.6 (73.9).

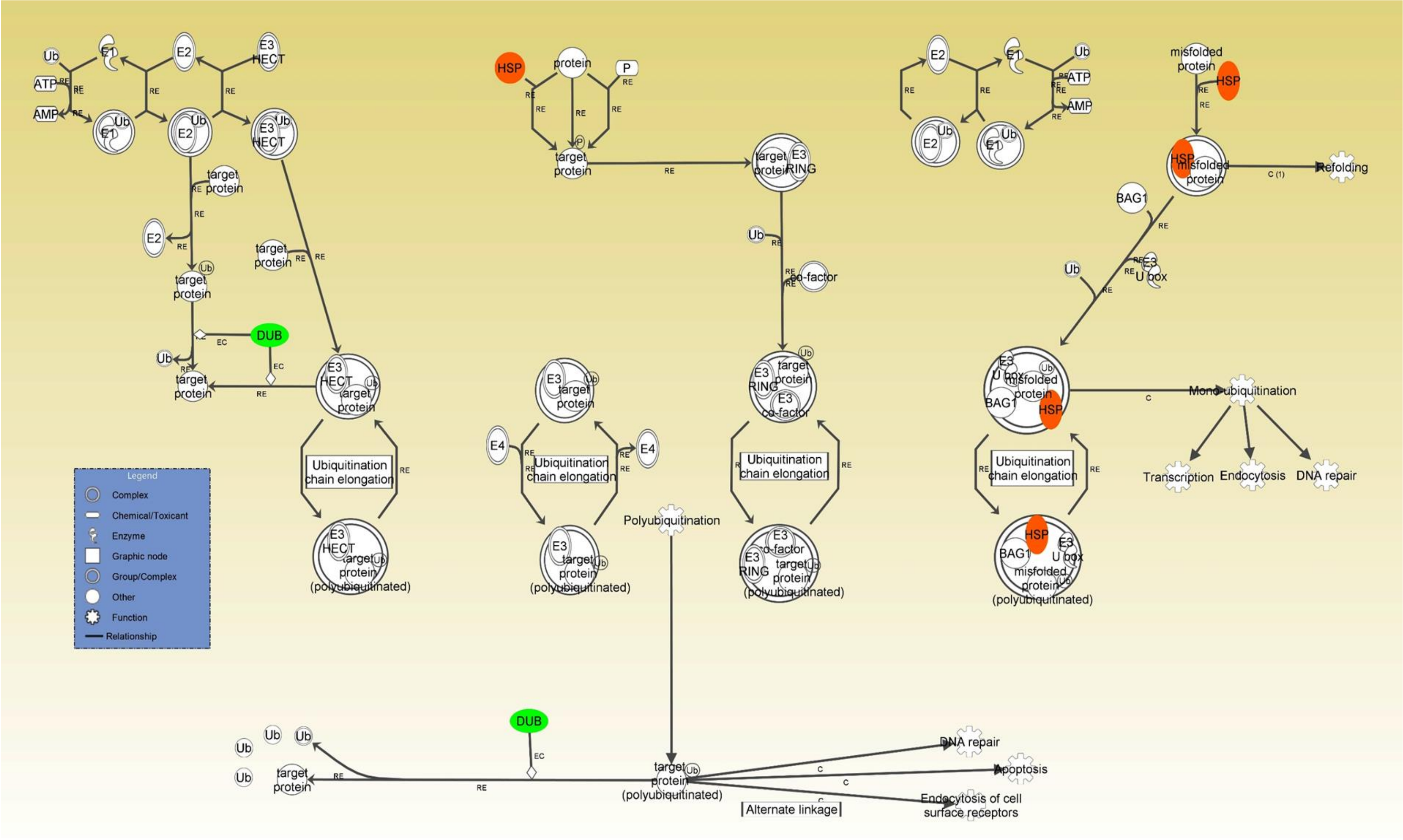

Supplement: Supplementary file 1 — Supplementary Tables and Figures [file 41598_2017_5266_MOESM1_ESM.pdf]
